# Assessment of the DNA barcode libraries for the study of the poorly-known rove beetle (Staphylinidae) fauna of West Siberia

**DOI:** 10.3897/BDJ.11.e115477

**Published:** 2023-12-20

**Authors:** Valeria Krivosheeva, Alexey Solodovnikov, Aleksandr Shulepov, Darya Semerikova, Anastasiya Ivanova, Maria Salnitska

**Affiliations:** 1 X-BIO Institute, University of Tyumen, Tyumen, Russia X-BIO Institute, University of Tyumen Tyumen Russia; 2 Natural History Museum of Denmark, Copenhagen, Denmark Natural History Museum of Denmark Copenhagen Denmark; 3 MAGNIT information technologies, Krasnodar, Russia MAGNIT information technologies Krasnodar Russia

**Keywords:** biodiversity, BOLD, COI, ecology, GenBank, insects, taxonomy

## Abstract

Staphylinidae, or rove beetles, are one of the mega-diverse and abundant families of the ground-living terrestrial arthropods that is taxonomically poorly known even in the regions adjacent to Europe where the fauna has been investigated for the longest time. Since DNA barcoding is a tool to accelerate biodiversity research, here we explored if the currently-available COI barcode libraries are representative enough for the study of rove beetles of West Siberia. This is a vast region adjacent to Europe with poorly-known fauna of rove beetles and from where not a single DNA barcode has hitherto been produced for Staphylinidae. First, we investigated the faunal similarity between the rove beetle faunas of the climatically compatible West Siberia in Asia, Fennoscandia in Europe and Canada and Alaska in North America. Second, we investigated barcodes available for Staphylinidae from the latter two regions in BOLD and GenBank, the world's largest DNA barcode libraries. We conclude that the rather different rove beetle faunas of Fennoscandia, on the one hand and Canada and Alaska on the other hand, are well covered in both barcode libraries that complement each other. We also find that even without any barcodes originating from specimens collected in West Siberia, this coverage is helpful for the study of rove beetles there due to the significant number of widespread species shared between West Siberia and Fennoscandia and due to the even larger number of shared genera amongst all three investigated regions. For the first time, we compiled a literature-based checklist for 726 species of the West Siberian Staphylinidae supplemented by their occurrence dataset submitted to GBIF. Our script written for mining unique (i.e. not redundant) barcodes for a given geographic area across global libraries is made available here and can be adopted for any other regions.

## Introduction

**Figure 1.**
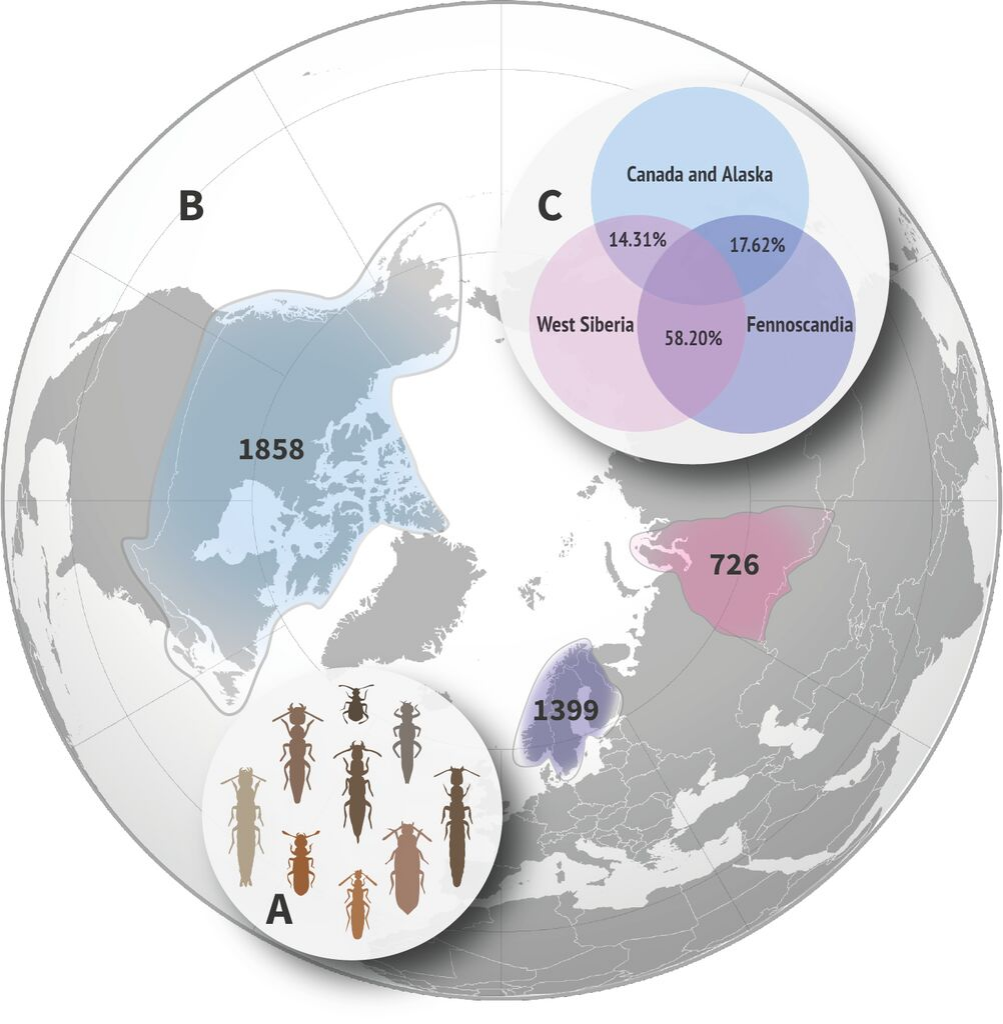
Rove beetles of the study areas and major facts about their faunas. **A** schematic sample of rove beetle diversity; **B** West Siberia (purple), Fennoscandia (violet) and Canada and Alaska (blue) with total number of Staphylinidae species registered in each of them; **C** similarity of the Staphylinidae faunas amongst the areas.

Rove beetles (Coleoptera, Staphylinidae) are the second largest family of living organisms (after weevils) (Fig. 1A). They are abundant in the majority of terrestrial habitats globally. Mostly, they are generalised predators or scavengers independent from the single impact of particular factors like, for example, distribution of the host plants or a particular prey species (Herman 2001, Thayer 2016, Betz et al. 2018). Therefore, rove beetles are a good proxy group to explore macroecological and biogeographic patterns. Staphylinids currently comprise nearly 67,000 described species globally (Newton (2022) in the Catalogue of Life at https://www.catalogueoflife.org) and they are always present in good numbers in various biodiversity assessment samples of the ground-based substrates (Betz et al. 2018).

To efficiently use rove beetles or other comparably diverse organismal groups in biodiversity studies, one needs, as a minimum, to quickly and precisely identify multiple species in the large samples. This is a daunting task requiring expensive, time-consuming and, nowadays, rare taxonomic expertise. DNA-based techniques, especially barcoding, now can serve for overcoming such an impediment. In the course of the last two decades, barcoding grew into an important and popular tool of biodiversity exploration and monitoring with sound prospects to increase its impact in the future (Schlick-Steiner et al. 2010, Taberlet et al. 2012, Yu et al. 2012, Liu et al. 2019, Grant et al. 2021). Growth of the DNA barcoding into a multi-purpose research tool led to the formation of the barcode reference libraries. Barcode of Life Data Systems (BOLD; Ratnasingham and Hebert (2007)) is the largest database of this kind that contains more than 12.4 million DNA barcodes (Grant et al. 2021) and is constantly growing. Another is the GenBank database that contains more than 3.5 million COI barcodes (Sayers et al. 2021). Performance and degree of completeness of these databases for various taxa, regions and applications vary and have been subject of investigations (for example, Gaytán et al. (2020), Piemontese et al. (2020), Cheng et al. (2023)).

Here, we want to explore if BOLD and GenBank already gained critical mass of data to study Staphylinidae in West Siberia, i.e. a region where we know that the fauna is poorly known and DNA barcodes were never sampled. In general, we know that, for well investigated regions like, for example, Central Europe or Canada, many beetle specimens can be quickly and reliably identified by its DNA barcode alone already (Hendrich et al. 2014, Gwiazdowski et al. 2015, Schmidt et al. 2015, Hawlitschek et al. 2016, Hebert et al. 2016, Pentinsaari et al. 2019). For the more poorly explored taxonomic groups and regions, i.e. the frontline of biodiversity exploration, we remain far from such an ideal situation, with much to be done. Obviously, the use of the DNA barcoding is less straightforward under conditions of limited data and knowledge. With respect to north temperate Staphylinidae, regions vary from very well-studied like Central and Northern Europe, through reasonably well-explored with knowledge gaps like USA and Canada to poorly known like Siberia. Since many rove beetle species have the latitudinally and longitudinally very extensive distribution ranges across the Holarctic or its sub-regions (Herman 2001), we should expect some overlap amongst local faunas as, for example, is shown by the recently revised genus *Quedius* (Salnitska and Solodovnikov 2019, Hansen et al. 2022). Thus, under conditions of such an overlap, we want to explore how much the currently available DNA barcode libraries accumulated for regions with better known faunas of Staphylinidae can be applied for the study of their poorly-known faunas.

## Study design

We select West Siberia as a target region with poorly-known rove beetle fauna; as well as Fennoscandia in Europe and Canada and Alaska in North America (Fig. 1B), i.e. the regions with, expectedly, very or somewhat similar faunas with West Siberia, respectively. These comparative regions were also chosen because it was relatively easy to generate their comprehensive faunistic lists of Staphylinidae.

West Siberia is a large part of Eurasia with rather clear boundaries defined by topography. From the west, it is outlined by the eastern foothills of the Ural Range, in the south by South Siberian Mountains and Kazakh Uplands, in the east by the Yenisei Ridge and the Central Siberian Plateau and in the north by the Kara Sea. The total area of West Siberia is almost 3 million km², the length from north to south is almost 2500 km, from west to east about 1900 km. West Siberia is a flat lowland spread from tundra in the north through extensive taiga forest to steppe in the south (Gvozdeckiy and Mihalov 1978). Due to its large and hardly accessible terrain, rove beetles and other arthropods of West Siberia are poorly studied. However, we were able to pull together all scattered information on rove beetles of that region for this study, as described below.

Fennoscandia (Ramsay 1898) is outlined by the Baltic Sea in the south, the North Sea in the west, the Norway and the Barents Seas in the North and the White Sea and the East European Plain in the east. The total area is 1.88 million km². Its landscapes vary from plains adjoining the Baltic Sea to mountains adjoining the Norway Sea (Kulikov and Kulikova 2013). Its natural zones span from tundra in the north through boreal to broad-leaved forests in the south (Golubyatnikov and Mammarella 2018). The Fennoscandian biodiversity is intensively studied in many ways (Bjørnstad et al. 1995, Angerbjörn et al. 2001, Kozlov et al. 2022). Due to historical reasons, the Fennoscandian rove beetle fauna (Silfverberg 2011) is amongst the best investigated in Europe (Benick 1934, Hansen et al. 1939, Herman 2001, Hansen et al. 2018).

Contrary to West Siberia and Fennoscandia, Canada and Alaska comprise a much larger area, outlined by the shores of the Atlantic Ocean (Baffin Bay and Labrador Sea) in the east, the Arctic Ocean (Beaufort Sea) in the north and the Pacific Ocean (Bering Strait) in the west (Baulig 1936). Its southern boundary is not defined with the relief, but separated by biogeographic patterns as Canada and Alaska almost match with the Arctic subregion of the Nearctic Region (Escalante et al. 2021). The total area of Canada and Alaska is almost 12 million km² (Hall et al. 2023, Lynch and Miller 2023). Its natural zones stretch from tundra in the north to mixed forests in the south (Larocque et al. 2006, Johansson et al. 2013). It is possible to extract a reliable list of species of rove beetles for this area due to existing literature compilations (Bousquet et al. 2013).

We mine barcodes in BOLD and GenBank for each of these three study regions in order to know: (1) how complete these databases are for their rove beetle faunas; (2) which of both databases is more complete for Staphylinidae as a whole or for the chosen regional faunas, how do they overlap and what is the difference between them; (3) which database is better to use and for which purposes. For seeking answers to these questions we: (1) compile a species list of rove beetles for each of three target regions; (2) compare their faunas based on these species lists; (3) explore both BOLD and GenBank for the presence and diversity of the COI barcodes for the listed species, for all three target geographic regions; (4) explore an overlap and peculiarities of each database in order to eliminate duplication of information and focus on the unique barcodes of each species; and (5) reflect on the observed patterns.

## Material and Methods

### Taxonomic species lists for Staphylinidae of the target regions and their overlap

A list of Staphylinidae species fauna of West Siberia was compiled from references published before 2023. For that, occurrences of species recorded in West Siberia were databased using the EarthCape Biodiversity Database Platform (Meyke 2019) software via Windows Client. These occurrences were obtained from scientific papers using various sources including Google Scholar (https://scholar.google.ru) and e-library (https://elibrary.ru). All occurrence data from EarthCape, stored at the server of the Zoological Institute of the Russian Academy of Science in St. Petersburg, were exported into a taxonomic list of Staphylinidae species of West Siberia (Suppl. material 1).

A list of Fennoscandian Staphylinidae contains species that occur in Finland, Sweden, Norway and north-western part of Russia. It is based on the Fennoscandian catalogue of beetles (Silfverberg 2011), recent research on the soil fauna of north-western Russia (Kozlov et al. 2022) and occurrences recorded in GBIF (GBIF.org 2023a).

A list of Staphylinidae species fauna of Canada and Alaska is based on the Checklist of beetles of that region (Bousquet et al. 2013) and GBIF occurrence data (GBIF.org 2023b). All three regional species lists, used as input data in our study, are provided in Suppl. material 2.

Species level taxonomy and higher classification of Staphylinidae were used according to Schülke and Smetana (2015) and Newton (2022). Newton (2022) on-line database in the Catalogue of Life (https://www.catalogueoflife.org) accounts for the new division of Tachyporinae into Tachyporinae propria and Mycetoporinae (Yamamoto 2021), but it does not follow the resurrection of the subfamily Xantholininae from Staphylininae by Żyła and Solodovnikov (2020). We consider Xantholininae in the rank of the subfamily as in Żyła and Solodovnikov (2020). We did not include Silphinae in our study as it was ranked as a subfamily of Staphylinidae only recently (Cai et al. 2022), which is not reflected yet in many catalogues or databases which makes automatic extraction of data more complex.

We used the Czekanowski–Sørensen index (Czekanowski 1913, Sørensen 1948, Pesenko 1982) and Jaccard index (Jaccard 1901, Costa 2021) for the pairwise comparison of the faunal composition of the target study regions. Computing the index was performed using a Python script (available at https://github.com/alexandershulepov/assessment-of-the-dna-barcode-libraries).

### Mining COI barcode sequences from BOLD and GenBank

All available COI barcodes and their metadata for species that occur in three target regions were obtained from BOLD (https://boldsystems.org) and GenBank (https://www.ncbi.nlm.nih.gov/genbank/). From BOLD, data were obtained by using a python script developed for this paper and made available at https://github.com/alexandershulepov/assessment-of-the-dna-barcode-libraries. The script downloaded all needed information about barcodes for each species in the form of a TSV table. In addition, this script enabled us to perform some steps of the analysis of the downloaded data (see Data analysis section below).

From GenBank, barcodes were manually downloaded and saved in the GenBank (gb) data format. Sequences were searched for each species by “*species name* Cytochrome oxidase subunit I” search query in the GenBank Nucleotide database.

### Data analysis

For the major steps of data analysis, we used the same python script that we created for obtaining barcode sequences (see above). For the barcodes from BOLD, it computed their total number for each species and their numbers from specimens originating in each of the target geographical regions. All this information was generated into CSV tables. For analysing barcodes from GenBank, first we manually downloaded sequences which afterwards served as an input for this script. For the barcodes from GenBank, using some functions of the script, we parsed GenBank files into the taxonomic name, the unique code (‘version’) and the country where a beetle for sequencing was collected. To ensure that our analyses will be based on the same barcoding fragment of the COI-5P gene, first, we eliminated COI-3P sequences obtained with Pat (TL2-N-3014) and Jerry (Cl-J-2183) primers that were originally downloaded together with the proper barcoding fragment. This step was required due to the fact that GenBank does not provide clear information about the exact gene region. This was done with the script function. The COI fragment amplified by the Pat and Jerry primer pair, mainly for phylogenetic purposes, has almost no overlap with the barcode region (Simon et al. 1994). Second, from the originally downloaded pool, we disregarded 44 sequences that had no information pointing to an exact gene region, because, potentially, they could also represent non-barcoding regions. Since some barcodes are doubled in both BOLD and GenBank, we added to the script a function that determines cases of such duplication via comparing barcode’s unique IDs and counts only original one from the pair. As an output, the script gave tables with the amount of barcodes per species (Suppl. material 3). Based on the summary tables, all further calculations were conducted using Microsoft Excel tools and functions. Illustrations were prepared in Miro (miro.com).

It should be noted that results have a margin of error due to ambiguous data in barcode libraries that may not be up-to-date with the taxonomy and sometimes not taking into account synonymy. For example, the barcode of species identified as *Athetacampbelli* (Lohse, 1990) in GenBank was added there under the ID number UAMIC2628-15 from BOLD. At the same time, the sequence with this ID in BOLD is identified as *Athetaallocera* Eppelsheim, 1893. Both names are synonyms and refer to the same species with the valid name *Athetaallocera* Eppelsheim, 1893. The same situation is with *Gnypetaminuta* Klimaszewski et Webster, 2008, a synonym of *Dasygnypetavelata* (Erichson, 1837); *Oxypodagrandipennis* (Casey, 1911), a synonym of *Oxypodasylvia* Casey, 1906; *Lathrobiumsimplex* LeConte, 1880, a synonym of *Lathrobiumfauveli* Duvivier, 1883; and other similar examples. It appears that, in BOLD, the taxonomy is being updated and more up-to-date than in GenBank. Additionally, the species checklists that we generated for the target regions and used for mining barcodes may contain a few fossil species that were not eliminated and for which barcodes are, in fact, absent not because they were not sequenced, but because they are not possible in principle.

## Results

### Species list of rove beetles of West Siberia

Overall, we found 27 publications with reasonably reliable, not outdated species identifications as a source to compile a species checklist of rove beetles that were hitherto recorded from West Siberia. Amongst them, the Palaearctic Catalogue (Schülke and Smetana 2015) lists 591 species of Staphylinidae that are recorded for West Siberia. These catalogue records only state that a species occurs somewhere in West Siberia (coded as WS). These catalogue records together with the other publications, that are mostly Russian faunistic studies, give 726 species of Staphylinidae hitherto recorded in West Siberia. Records from the faunistic or systematic literature are more detailed than in the Palaearctic Catalogue and mainly report a species for West Siberia either from particular localities or at least from certain geographic or administrative areas of this region. The full checklist of the fauna with corresponding references for all species is provided in Suppl. material 1 (and sent to GBIF).

### West Siberian rove beetle fauna in comparison with Fennoscandia and Canada and Alaska

The currently recorded fauna of Fennoscandia comprises 1399 species of Staphylinidae; Canada and Alaska – 1858 species; West Siberia – 726 species (Fig. 1B).

According to the Czekanowski–Sørensen index and expectedly for the same continent, at the species level, the Staphylinidae fauna of Fennoscandia is rather similar to the West Siberian fauna (58.20%). Similarity of the Canadian and Alaskan rove beetle faunas with either Fennoscandian (17.62%) or West Siberian (14.31%) faunas is much lower (Fig. 1C). These values are lower for Jaccard similarity, but higher for both indices if the faunal similarity is computed for genera (for comparison, see Table 1). The faunal similarity between Eurasian (measured by the Fennoscandian and West Siberian faunas) and North American (measured by Canadian and Alaskan fauna) continents is 17.64% according to the Czekanowski–Sørensen index and 9.67% according to the Jaccard index.

### Barcode coverage for species

**Figure 2.**
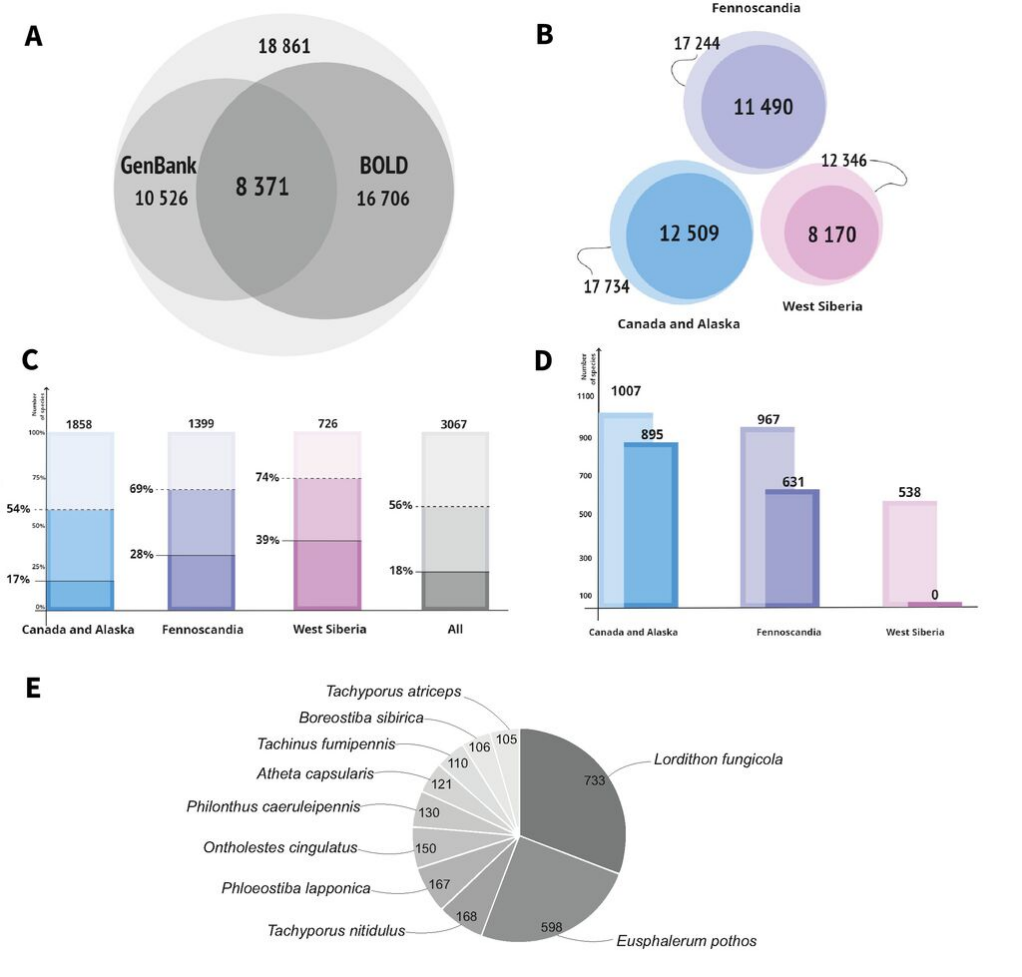
Statistical patterns of the COI barcodes of Staphylinidae available from GenBank and BOLD databases to estimate their utility for the study of West Siberian fauna. **A** Number of downloaded COI barcodes from Bold and GenBank (smaller circles), an overlap formed by the same barcodes duplicated in both databases and total number of unique (not overlapping) barcodes in both libraries (largest circle); **B** Numbers of unique (not duplicated in Bold and GenBank) barcodes for each of three study regions (smaller darker circles) in comparison with their total numbers of barcodes from both libraries (larger pale circles); **C** Percentage of species from the total number of species in the fauna (numbers on top of the bars) with with at least one (dotted line) and 10 (solid line) unique barcodes in each of the study regions and in all regions combined; **D** Numbers of species with at least one barcode sequenced from specimens collected anywhere (pale bars) and from specimens collected within each of the study regions (darker bars); **E** Species of rove beetles barcoded the most with their respective numbers of barcodes available.

In total, 27232 barcodes were mined from BOLD and GenBank altogether for all species from all three target geographical regions. Amongst them, 8371 are sequences submitted to both GenBank and BOLD (Fig. 2A, an overlap area amongst smaller circles). Therefore, only 18862 barcodes out of 27232 are unique (i.e. not duplicated in both libraries (Fig. 2A, largest circle). Eliminating these duplicates from any further statistics, we found 12509 barcodes for Staphylinidae of Canada and Alaska (amongst them, 1021 from GenBank and 6263 from BOLD not doubling each other), 11490 for Fennoscandia (1903 from GenBank and 3833 from BOLD not doubling each other) and 8170 for West Siberia (1500 from GenBank and 2494 from BOLD not doubling each other) (Fig. 2B). As can be seen from Fig. 2C, the fauna is greatest in Canada and Alaska and smallest in West Siberia, but the percentage of species for which barcodes are available is the reverse: it is the highest in West Siberia (74%), followed by Fennoscandia (69%) and then Canada and Alaska (54%). As shown in the Fig. 2C in the rightmost bar, based on the total pool of data from both BOLD and GenBank, 1722 (56%) species from all regions have at least one barcode, i.e. they have at least one specimen from anywhere sequenced. The same bar illustrates a much smaller number of species represented by several barcoded specimens. For example, 550 (18%) species have more than 10 barcodes. Again, West Siberia has the highest share of species represented by more than 10 barcodes (39%), followed by Fennoscandia (28%) and Canada and Alaska (17%) (Fig. 2C).

However, when we take the geographic origin of the barcoded specimens into consideration (Fig. 2D), out of 538 species with the available barcodes in West Siberia, there is not a single barcode amongst them that would come from material collected there. All West Siberian species with the available barcodes are, in fact, more or less widespread species that were sampled for barcoding elsewhere. On the contrary (Fig. 2D), out of 967 barcoded species in Fennoscandia 631 (45% of the fauna and 65% of the barcoded species), these have barcodes generated from specimens collected within the borders of Fennoscandia itself; and out of 1092 barcoded species from Canada and Alaska 895 (48% of the fauna and 89% of the barcoded species), these have barcodes generated from specimens collected within the borders of that region, respectively.

Amongst the Staphylinidae species with a large number of barcodes (Fig. 2E), several were sequenced many more times than others. For instance, *Bolitobiusfungicola* (Campbell, 1982) (= Lordithon fungicola Cambell, 1982) is represented by 733 barcodes, *Eusphalerumpothos* (Mannerheim, 1843) by 598 barcodes, *Tachyporusnitidulus* (Fabricius, 1781) by 168 barcodes, *Phloeostibalapponica* (Zetterstedt, 1838) by 167 barcodes, *Ontholestescingulatus* (Gravenhorst, 1802) by 150 barcodes etc.

### BOLD versus GenBank

BOLD has the largest pool of barcodes for rove beetle species from our target regions. For Canada and Alaska, BOLD contains 878 species with barcodes from this territory and GenBank only 446. For Fennoscandia, BOLD provides 619 species with barcodes from that area and GenBank 535 species. None of the databases provides barcodes from specimens collected in West Siberia.

## Discussion and Conclusion

Our data exploration stresses the poor state of knowledge of the Staphylinidae beetles of West Siberia, which is an impediment for using this common and ubiquitous group of the soil macroinvertebrates as a proxy for exploring ground patterns of biodiversity in Eurasia and globally. Contrary to the comprehensive faunal lists of Fennoscandia or Canada and Alaska, both made available by single comprehensive summary resources, the West Siberian faunal list had to be compiled from 27 scattered publications. These publications with reasonably reliable, not outdated species identifications, were filtered from a larger pool of older publications where many identifications were ambiguous. The Palaearctic Catalogue (Schülke and Smetana 2015) is the most significant source that lists 591 species of Staphylinidae for West Siberia, albeit it does not provide the data its records are based on. A high proportion of Staphylinidae species (206 of 726) are known for that region from no more than these catalogue records without any precise baseline data. Most of the georeferenced West Siberian records originate from the southern, better investigated, areas of this region.

Based on the published data, the rove beetle fauna of Canada and Alaska is the largest by the number of species, followed by the fauna of Fennoscandia and then West Siberian fauna. This seems natural as Canada and Alaska cover a territory the largest of all three compared regions. Interestingly, Fennoscandia, an area which is several times smaller than Canada and Alaska, has the rove beetle fauna that is smaller than the fauna of the latter much larger area, only by a few hundred species. This can be explained by the combined effect of two factors. Firstly, the Fennoscandian fauna is much better explored compared to the fauna of Canada and Alaska and, secondly, the European rove beetle fauna is rather species-rich. The number of Staphylinidae species in West Siberia, an area which is geographically larger than Fennoscandia, is about two times smaller than the number of species in the former smaller area. Again, this can be explained by the much poorer degree of our knowledge of the West Siberian fauna, as well as perhaps by the naturally poorer fauna of the latter with more continental and harsh climate and with more homogeneous flat relief. With the future faunal explorations of all study areas, we expect significant increase of rove beetle species for Canada and Alaska and for West Siberia.

Expectedly, the best explored Fennoscandian rove beetle fauna is also the best barcoded compared to other two regions. In fact, it is barcoded to an impressively high degree, where many more than half of the species in the fauna have at least one barcode available from somewhere. One must be aware, however, that even for the well-barcoded Fennoscandian fauna, only nearly a half (45%) of the species in the fauna have barcodes generated from specimens collected within the borders of Fennoscandia. For the much less explored rove beetle fauna of West Siberia, the percentage of species with the available barcodes is even higher than for Fennoscandia, even though none of these barcodes comes from specimens collected in West Siberia itself. We explain this firstly by the high faunal similarity between Fennoscandia and West Siberia and, secondly, by the fact that the faunal list of the much poorer explored fauna of West Siberia is dominated by the common West- or Transpalaearctic widespread species which also occur elsewhere in Europe including Fennoscandia. All barcodes conspecific with species found in West Siberia were generated from specimens collected elsewhere, mainly in Europe. We foresee that further exploration of the West Siberian rove beetle fauna will add a percentage of Asian species for which barcode data are sparse. Even though the percentage of species with the available barcodes in Canada and Alaska is substantially lower than in West Siberia and Fennoscandia, it is noteworthy that barcodes are already available for more than a half of the fauna of this immensely large territory and more than a half of these barcodes are generated from specimens collected there.

BOLD and GenBank contain a number of identical barcodes, i.e. ca. one quarter of their data is doubled between both databases because the same barcode was deposited in both of them. For the majority of their barcodes, however, both databases are unique and, thus, they complement each other. For example, GenBank has approximately two thousand barcodes that appear only there. BOLD, on the contrary, has around eight thousand barcodes that belong only to this database. This could be explained by the BOLD initiatives of collecting barcodes from GenBank and, vice versa, GenBank collects data from BOLD. Most of barcodes relevant for rove beetle species of Fennoscandia and West Siberia come from the DNA barcoding initiatives launched for beetles in Central Europe, especially in Germany (Hendrich et al. 2014, Rulik et al. 2017) or in Northern Europe (Pentinsaari et al. 2014). Some researchers originally upload their data to BOLD (Hendrich et al. 2014, Rulik et al. 2017) and some to GenBank (Pentinsaari et al. 2014). However, the majority, especially in North America, is now leaning to upload barcodes to BOLD, even though some concerns were raised about BOLD not making all its tools publicly available (Meier et al. 2022). Large barcoding projects for beetles of Canada and Alaska (Hebert et al. 2016, deWaard et al. 2019), almost all stimulated by BOLD, were initiated in Canada, a home country for the BOLD headquarters. Naturally, most of the American barcodes are deposited in BOLD.

Substantial overlap in species composition between the West Siberian and Fennoscandian rove beetle faunas and some overlap between northern Eurasian and northern North American faunas, due to numerous more or less widespread species, greatly increases the available pool of barcodes for particular species due to extralimital barcodes. This is especially obvious for the West Siberian rove beetle fauna, for which all available barcodes are extralimital, i.e. mainly coming from specimens collected in Europe. The badly-needed inventory of the West Siberian rove beetle fauna can and should be facilitated by using even such extralimital DNA barcodes to quickly separate potential new species from the known ones. More efficient identification of species via barcoding will include discovery of the hitherto unrecognised molecular variants within the widespread variable morphospecies. It is, in fact, impressive that the available barcode resources can be used for that despite a complete lack of barcodes from West Siberia as such. Noteworthy is the lack of the taxonomic bias amongst the available barcodes. There is no bias towards a more comprehensive barcoding of the larger or otherwise more popular beetles, albeit, for example, the better known and larger, more commonly collected Staphylinidae are indeed relatively well barcoded, while small and taxonomically poorer known are barcoded to a lesser degree. It should be noted, however, that the slow pace of the taxonomic updates of the public barcode databases can cause some gaps or confusion in the research. For example, none of the databases includes Silphinae as a subfamily of Staphylinidae or one database may be more updated than the other with respect to taxonomy. Both GenBank and BOLD should be used as a source of barcode data and data from both sources should be critically checked as far as possible.

## Supplementary Material

50D33871-B98E-5438-BE58-D6C59BC55982 10.3897/BDJ.11.e115477.suppl1 10424616 Supplementary material 1 Checklist of Staphylinidae species from West Siberia Data type Checklist Brief description Species list of Staphylinidae from West Siberia with references from which the records come. File: oo_932338.docx https://binary.pensoft.net/file/932338 Krivosheeva V, Solodovnikov A, Shulepov A, Semerikova D, Ivanova A, Salnitska M

9E516CF5-FBFC-553B-BAB7-152CF5ED6FF1 10.3897/BDJ.11.e115477.suppl2 10424624 Supplementary material 2 Lists of Staphylinidae species from three study regions Data type Species list Brief description Species lists of Staphylinidae from Fennoscandia, West Siberia, as well as from Canada and Alaska, three lists in total. File: oo_947612.xlsx https://binary.pensoft.net/file/947612 Krivosheeva V, Solodovnikov A, Shulepov A, Semerikova D, Ivanova A, Salnitska M

7AC8460B-4475-5832-9D0A-0449B6109B3B 10.3897/BDJ.11.e115477.suppl3 Supplementary material 3 Output script data Data type Script output Brief description Output data from script (https://github.com/alexandershulepov/assessment-of-the-dna-barcode-libraries), contains rove beetle species lists of the target regions and amounts of COI barcodes per species. File: oo_947613.xlsx https://binary.pensoft.net/file/947613 Krivosheeva V, Solodovnikov A, Shulepov A, Semerikova D, Ivanova A, Salnitska M

## Figures and Tables

**Table 1.** Similarity of the Staphylinidae faunas of the study areas for species and genus levels, based on Jaccard index and Czekanowski–Sørensen index.

	Species level		Genus level	
	Jacard index	Czekanowski–Sørensen index	Jacard index	Czekanowski–Sørensen index
WS-CA	7.71%	14.31%	36.22%	51.18%
WS-FS	41.06%	58.20%	59.52%	74.46%
FS-CA	9.66%	17.62%	43.40%	60.44%
