# Supplementary material for: Assessment of the DNA barcode libraries for the study of the poorly-known rove beetle (Staphylinidae) fauna of West Siberia
Source: Biodivers Data J. 2023 Dec 20;11:e115477. doi: 10.3897/BDJ.11.e115477 (PMC10755746; doi:10.3897/BDJ.11.e115477)
Supplement: Supplementary material 1 — Checklist of Staphylinidae species from West Siberia [file bdj-11-e115477-s001.docx]

**Subfamily *Aleocharinae* Fleming, 1821**

**Tribe** *Aleocharini* Fleming, 1821

**Genus** *Aleochara* Gravenhorst, 1802

*Aleochara bilineata* Gyllenhal, 1810

Kemerovo Prov. (Babenko, 1982); Tomsk Prov. (Babenko, 1982); WS (Schülke, Smetana, 2015).

*Aleochara binotata* Kraatz, 1856

Tyumen Prov. (Bukhkalo et al., 2011, 2012); WS (Schülke, Smetana, 2015).

*Aleochara bipustulata* (Linnaeus, 1760)

Kemerovo Prov. (Babenko, 1982); Tomsk Prov. (Babenko, 1982); WS (Schülke, Smetana, 2015).

*Aleochara brevipennis* Gravenhorst, 1806

Tyumen Prov. (Bukhkalo et al., 2011, 2012); WS (Schülke, Smetana, 2015).

*Aleochara curtula* (Goeze, 1777)

Kemerovo Prov. (Babenko, 1982); Tomsk Prov. (Babenko, 1982); (Schülke, Smetana, 2015).

*Aleochara diversa* (J. Sahlberg, 1876)

WS (Schülke, Smetana, 2015).

*Aleochara fumata* Gravenhorst, 1802

Khanty-Mansi Distr. (Filippov, 2011); Tyumen Prov. (Bukhkalo et al., 2011, 2012); WS (Schülke, Smetana, 2015).

*Aleochara funebris* Wollaston, 1864

WS (Schülke, Smetana, 2015).

*Aleochara laevigata* Gyllenhal, 1810

Tyumen Prov. (Bukhkalo et al., 2011, 2012); WS (Schülke, Smetana, 2015).

*Aleochara lanuginosa* Gravenhorst, 1802

WS (Schülke, Smetana, 2015).

*Aleochara lepidoptera* Bernhauer, 1901

WS (Schülke, Smetana, 2015).

*Aleochara lygaea* Kraatz, 1862

WS (Schülke, Smetana, 2015).

*Aleochara milleri* Kraatz, 1862

Kemerovo Prov. (Babenko, 1982); Tomsk Prov. (Babenko, 1982); WS (Schülke, Smetana, 2015).

*Aleochara moerens* Gyllenhal, 1827

Khanty-Mansi Distr. (Filippov, 2011); Tyumen Prov. (Bukhkalo et al., 2011, 2012); WS (Schülke, Smetana, 2015).

*Aleochara morion* Gravenhorst, 1802

WS (Schülke, Smetana, 2015).

*Aleochara parvicornis* Fauvel, 1900

WS (Schülke, Smetana, 2015).

*Aleochara pernigra* Schubert, 1906

WS (Schülke, Smetana, 2015).

*Aleochara spissicornis* Erichson, 1839

Tyumen Prov. (Bukhkalo et al., 2011, 2012); WS (Schülke, Smetana, 2015).

*Aleochara tristis* Gravenhorst, 1806

WS (Schülke, Smetana, 2015).

*Aleochara villosa* Mannerheim, 1830

Tyumen Prov. (Bukhkalo et al., 2011, 2012); WS (Schülke, Smetana, 2015).

**Genus** *Amarochara* Thomson, 1858

*Amarochara umbrosa* (Erichson, 1837)

WS (Schülke, Smetana, 2015).

**Tribe** *Athetini* Casey, 1910

**Genus** *Acrotona* Thomson, 1859

*Acrotona aterrima* (Gravenhorst, 1802)

Tyumen Prov. (Bukhkalo et al., 2011, 2012); WS (Schülke, Smetana, 2015).

*Acrotona convergens* (A.Strand, 1958)

Tyumen Prov. (Bukhkalo et al., 2011, 2012); WS (Schülke, Smetana, 2015).

*Acrotona exigua* (Erichson, 1837)

WS (Schülke, Smetana, 2015).

*Acrotona obfuscata* (Gravenhorst, 1802)

WS (Schülke, Smetana, 2015).

*Acrotona parvula* (Mannerheim, 1830)

WS (Schülke, Smetana, 2015).

*Acrotona pygmaea* (Gravenhorst, 1802)

Tyumen Prov. (Bukhkalo et al., 2011, 2012).

*Acrotona sylvicola* (Kraatz, 1856)

Tyumen Prov. (Bukhkalo et al., 2011, 2012); WS (Schülke, Smetana, 2015).

**Genus** *Alianta* Thomson, 1858

*Alianta curta* J. Sahlberg, 1880

WS (Schülke, Smetana, 2015).

**Genus** *Amidobia* Thomson, 1858

*Amidobia talpa* (Heer, 1841)

WS (Schülke, Smetana, 2015).

**Genus** *Amischa* Thomson, 1858

*Amischa analis* (Gravenhorst, 1802)

Tyumen Prov. (Bukhkalo et al., 2011, 2012); WS (Schülke, Smetana, 2015).

*Amischa bifoveolata* (Mannerheim, 1830)

WS (Schülke, Smetana, 2015).

*Amischa sahlbergi* (Eppelsheim, 1893)

WS (Schülke, Smetana, 2015).

**Genus** *Atheta* Thomson, 1858

*Atheta aeneipennis* (Thomson, 1856)

Tyumen Prov. (Bukhkalo et al., 2011); WS (Schülke, Smetana, 2015).

*Atheta altaica* Bernhauer, 1901

WS (Schülke, Smetana, 2015).

*Atheta amicula* (Stephens, 1832)

WS (Schülke, Smetana, 2015).

*Atheta aquatica* (Thomson, 1852)

WS (Schülke, Smetana, 2015).

*Atheta arctica* (Thomson, 1856)

Tyumen Prov. (Bukhkalo et al., 2011, 2012); Yamalo-Nenets Distr. (Olshvang, 1992); WS (Schülke, Smetana, 2015).

*Atheta atramentaria* (Gyllenhal, 1810)

WS (Schülke, Smetana, 2015).

*Atheta atricolor* (Sharp, 1869)

WS (Schülke, Smetana, 2015).

*Atheta banghaasi* Bernhauer, 1901

WS (Schülke, Smetana, 2015).

*Atheta basicornis* (Mulsant & Rey, 1952)

Tyumen Prov. (Bukhkalo et al., 2011, 2012); Khanty-Mansi Distr. (Krasutsky, 2005); WS (Schülke, Smetana, 2015).

*Atheta benickiella* Brundin, 1948

Tyumen Prov. (Bukhkalo et al., 2011, 2012); WS (Schülke, Smetana, 2015).

*Atheta boleticola* J. Sahlberg, 1876

Tyumen Prov. (Bukhkalo et al., 2011, 2012); WS (Schülke, Smetana, 2015).

*Atheta brachyptera* Brundin, 1944

Tyumen Prov. (Bukhkalo et al., 2011, 2012).

*Atheta britteni* Joy, 1907

Tyumen Prov. (Bukhkalo et al., 2011, 2012).

*Atheta canescens* (Sharp, 1869)

WS (Schülke, Smetana, 2015).

*Atheta castanoptera* (Mannerheim, 1830)

WS (Schülke, Smetana, 2015).

*Atheta cauta* (Erichson, 1837)

WS (Schülke, Smetana, 2015).

*Atheta celata* (Erichson, 1837)

WS (Schülke, Smetana, 2015).

*Atheta corvina* (Thomson, 1856)

WS (Schülke, Smetana, 2015).

*Atheta crassicornis* (Fabricius, 1792)

Tyumen Prov. (Bukhkalo et al., 2011, 2012); WS (Schülke, Smetana, 2015).

*Atheta dadopora* Thomson, 1867

Tyumen Prov. (Bukhkalo et al., 2011, 2012).

*Atheta debilis* (Erichson, 1837)

Tyumen Prov. (Bukhkalo et al., 2011, 2012); WS (Schülke, Smetana, 2015).

*Atheta depressicollis* (Fauvel, 1875)

Tyumen Prov. (Bukhkalo et al., 2011, 2012); WS (Schülke, Smetana, 2015).

*Atheta elongatula* (Gravenhorst, 1802)

Kemerovo Prov. (Babenko, 1982); Tomsk Prov. (Babenko, 1982); WS (Schülke, Smetana, 2015).

*Atheta fallaciosa* (Sharp, 1869)

Tyumen Prov. (Bukhkalo et al., 2011, 2012); WS (Schülke, Smetana, 2015).

*Atheta gagatina* (Baudi di Selve, 1848)

Tyumen Prov. (Bukhkalo et al., 2011, 2012).

*Atheta graminicola* (Gravenhorst, 1806)

Tyumen Prov. (Bukhkalo et al., 2011, 2012); WS (Schülke, Smetana, 2015).

*Atheta gyllenhalii* (Thomson, 1856)

Tyumen Prov. (Bukhkalo et al., 2011, 2012); WS (Schülke, Smetana, 2015).

*Atheta hygrobia* (Thomson, 1856)

Tyumen Prov. (Bukhkalo et al., 2011, 2012).

*Atheta hypnorum* (Kiesenwetter, 1850)

Tyumen Prov. (Bukhkalo et al., 2011, 2012); WS (Schülke, Smetana, 2015).

*Atheta laevana* (Mulsant & Rey,1852)

WS (Schülke, Smetana, 2015).

*Atheta lapponica* J. Sahlberg, 1876

Tyumen Prov. (Bukhkalo et al., 2011); WS (Schülke, Smetana, 2015).

*Atheta laticeps* (Thomson, 1856)

Tyumen Prov. (Bukhkalo et al., 2011, 2012); WS (Schülke, Smetana, 2015).

*Atheta laticollis* (Stephens, 1832)

Tyumen Prov. (Bukhkalo et al., 2011, 2012); WS (Schülke, Smetana, 2015).

*Atheta lativentris* J. Sahlberg, 1876

Tyumen Prov. (Bukhkalo et al., 2011, 2012).

*Atheta lebedevoensis* Likovsky, 1984

WS (Schülke, Smetana, 2015).

*Atheta lioglutoides* Bernhauer, 1901

WS (Schülke, Smetana, 2015).

*Atheta longicornis* (Gravenhorst, 1802)

Tyumen Prov. (Bukhkalo et al., 2011, 2012); WS (Schülke, Smetana, 2015).

*Atheta malleus* Joy, 1913

Tyumen Prov. (Bukhkalo et al., 2011, 2012); WS (Schülke, Smetana, 2015).

*Atheta melanaria* (Mannerheim, 1830)

WS (Schülke, Smetana, 2015).

*Atheta melanocera* (Thomson, 1856)

Tyumen Prov. (Bukhkalo et al., 2011, 2012); WS (Schülke, Smetana, 2015).

*Atheta nigritula* (Gravenhorst, 1802)

Tyumen Prov. (Bukhkalo et al., 2014).

*Atheta occulta* (Erichson, 1837)

WS (Schülke, Smetana, 2015).

*Atheta oreophila* (Bernhauer, 1901)

WS (Schülke, Smetana, 2015).

*Atheta palleola* (Erichson, 1837)

Tyumen Prov. (Bukhkalo et al., 2011, 2012).

*Atheta pallidicornis* (Thomson, 1856)

Tyumen Prov. (Bukhkalo et al., 2011, 2012); WS (Schülke, Smetana, 2015).

*Atheta paracrassicornis* Brundin, 1954

Tyumen Prov. (Bukhkalo et al., 2011, 2012).

*Atheta pilicornis* (Thomson, 1852)

Tyumen Prov. (Bukhkalo et al., 2011, 2012).

*Atheta* *pittionii* Scheerpeltz, 1950

Tyumen Prov. (Bukhkalo et al., 2014)

*Atheta polaris* (Bernhauer, 1901)

WS (Schülke, Smetana, 2015).

*Atheta ripicola* Hanssen, 1932

Khanty-Mansi Distr. (Filippov, 2011); WS (Schülke, Smetana, 2015).

*Atheta sodalis* (Erichson, 1837)

Tyumen Prov. (Bukhkalo et al., 2011, 2012); WS (Schülke, Smetana, 2015).

*Atheta sordidula* (Erichson, 1837)

WS (Schülke, Smetana, 2015).

*Atheta strandiella* Brundin, 1954

Tyumen Prov. (Bukhkalo et al., 2011, 2012); WS (Schülke, Smetana, 2015).

*Atheta subtilis* (W. Scriba, 1866)

Khanty-Mansi Distr. (Filippov, 2011); Tyumen Prov. (Bukhkalo et al., 2011, 2012).

*Atheta terminalis* (Gravenhorst, 1806)

WS (Schülke, Smetana, 2015).

*Atheta tmolosensis* Bernhauer, 1940

Tyumen Prov. (Bukhkalo et al., 2011).

*Atheta vaga* (Heer, 1839)

Tyumen Prov. (Bukhkalo et al., 2011, 2012); WS (Schülke, Smetana, 2015).

*Atheta volans* W. Scriba, 1859

Tyumen Prov. (Bukhkalo et al., 2014).

**Genus** *Boreophilia* G. Benick, 1973

*Boreophilia eremita* (Rye, 1866)

Tyumen Prov. (Bukhkalo et al., 2011, 2012); WS (Schülke, Smetana, 2015).

*Boreophilia fusca* (C.R. Sahlberg, 1831)

WS (Schülke, Smetana, 2015).

*Boreophilia insecuta* (Eppelsheim, 1893)

WS (Schülke, Smetana, 2015).

*Boreophilia islandica* (Kraatz, 1857)

WS (Schülke, Smetana, 2015).

*Boreophilia subplana* (J. Sahlberg, 1880)

WS (Schülke, Smetana, 2015).

*Boreophilia vega* (Fenyes, 1920)

WS (Schülke, Smetana, 2015).

**Genus** *Boreostiba* Lohse, 1990

*Boreostiba sibirica* (Mäklin, 1880)

WS (Schülke, Smetana, 2015).

**Genus** *Dinaraea* Thomson, 1858

*Dinaraea aequata* (Erichson, 1837)

Khanty-Mansi Distr. (Krasutsky, 2005); Tyumen Prov. (Bukhkalo et al., 2011, 2012); Yamalo-Nenets Distr. (Krasutsky, 2005); WS (Schülke, Smetana, 2015).

*Dinaraea angustula* (Gyllenhal, 1810)

Tyumen Prov. (Bukhkalo et al., 2011, 2012); WS (Schülke, Smetana, 2015).

*Dinaraea linearis* (Gravenhorst, 1802)

Kemerovo Prov. (Babenko, 1982); Tomsk Prov. (Babenko, 1982); WS (Schülke, Smetana, 2015).

**Genus** *Dochmonota* Thomson, 1859

*Dochmonota clancula* (Erichson, 1837)

Tyumen Prov. (Bukhkalo et al., 2011, 2012); WS (Schülke, Smetana, 2015).

*Dochmonota rudiventris* (Eppelsheim, 1886)

Tyumen Prov. (Bukhkalo et al., 2011, 2012); WS (Schülke, Smetana, 2015).

**Genus** *Hydrosmecta* Thomson, 1858

*Hydrosmecta subtilissima* (Kraatz, 1854)

WS (Schülke, Smetana, 2015).

**Genus** *Liogluta* Thomson, 1858

*Liogluta alpestris* (Heer, 1839)

WS (Schülke, Smetana, 2015).

*Liogluta granigera* (Kiesenwetter, 1850)

WS (Schülke, Smetana, 2015).

*Liogluta longiuscula* (Gravenhorst, 1802)

WS (Schülke, Smetana, 2015).

*Liogluta micans* (Mulsant & Rey, 1851)

Tyumen Prov. (Bukhkalo et al., 2011, 2012).

*Liogluta microptera* Thomson, 1867

Tyumen Prov. (Bukhkalo et al., 2011, 2012).

*Liogluta turbida* (Eppelsheim, 1893)

WS (Schülke, Smetana, 2015).

**Genus** *Lypoglossa* Fenyes, 1918

*Lypoglossa lateralis* (Mannerheim, 1830)

Tyumen Prov. (Bukhkalo et al., 2014); WS (Schülke, Smetana, 2015).

**Genus** *Lyprocorrhe* Thomson, 1859

*Lyprocorrhe anceps* (Erichson, 1837)

WS (Schülke, Smetana, 2015).

**Genus** *Mocyta* Mulsant & Rey, 1874

*Mocyta amplicollis* (Mulsant & Rey, 1873)

Tyumen Prov. (Bukhkalo et al., 2011, 2012); WS (Schülke, Smetana, 2015).

*Mocyta clientula* (Erichson, 1839)

WS (Schülke, Smetana, 2015).

*Mocyta fungi* (Gravenhorst, 1806)

Tomsk Prov. (Babenko, Nuzhnykh, 2014); Tyumen Prov. (Bukhkalo et al., 2011, 2012, Vazhenina, 2019); WS (Schülke, Smetana, 2015).

*Mocyta orphana* (Erichson, 1837)

Tyumen Prov. (Bukhkalo et al., 2011, 2012); WS (Schülke, Smetana, 2015).

**Genus** *Notothecta* Thomson, 1858

*Notothecta flavipes* (Gravenhorst, 1806)

WS (Schülke, Smetana, 2015).

**Genus** *Schistoglossa* Kraatz, 1856

*Schistoglossa aubei* (Brisout de Barneville, 1860)

Tyumen Prov. (Bukhkalo et al., 2011, 2012).

*Schistoglossa gemina* (Erichson, 1837)

Tyumen Prov. (Bukhkalo et al., 2011, 2012); WS (Schülke, Smetana, 2015).

*Schistoglossa viduata* (Erichson, 1837)

Tyumen Prov. (Bukhkalo et al., 2011).

**Genus** *Tomoglossa* Kraatz, 1856

*Tomoglossa luteicornis* (Erichson, 1837)

WS (Schülke, Smetana, 2015).

**Tribe** *Autaliini* Thomson, 1859

**Genus** *Autalia* Leach, 1819

*Autalia longicornis Scheerpeltz, 1947*

Khanty-Mansi Distr. (Krasutsky, 2005);

**Tribe** *Falagriini* Mulsant & Rey, 1873

**Genus** *Anaulacaspis* Ganglbauer, 1895

*Anaulacaspis nigra* (Gravenhorst, 1802)

Tyumen Prov. (Bukhkalo et al., 2011, 2012); WS (Schülke, Smetana, 2015).

**Genus** *Falagria* Leach, 1819

*Falagria caesa* Erichson, 1837

WS (Schülke, Smetana, 2015).

*Falagria sulcatula* (Gravenhorst, 1806)

WS (Schülke, Smetana, 2015).

**Tribe** *Geostibini* Seevers, 1978

**Genus** *Aloconota* Thomson, 1858

*Aloconota gregaria* (Erichson, 1839)

Tyumen Prov. (Bukhkalo et al., 2011, 2012); WS (Schülke, Smetana, 2015).

**Genus** *Geostiba* Thomson, 1858

*Geostiba circellaris* (Gravenhorst, 1806)

Tyumen Prov. (Bukhkalo et al., 2011, 2012); Tomsk Prov. (Babenko, Nuzhnykh, 2014); WS (Schülke, Smetana, 2015).

**Tribe** *Gymnusini* Heer, 1839

**Genus** *Deinopsis* A. [H.] Matthews, 1838

*Deinopsis erosa* (Stephens, 1832)

Tyumen Prov. (Bukhkalo et al., 2011).

*Gymnusa* Gravenhorst, 1806

*Gymnusa atra* Casey, 1911

WS (Schülke, Smetana, 2015).

*Gymnusa brevicollis* (Paykull, 1800)

Tyumen Prov. (Bukhkalo et al., 2011, 2012); WS (Schülke, Smetana, 2015).

**Tribe** *Homalotini* Heer, 1839

**Genus** *Anomognathus* Solier, 1849

*Anomognathus cuspidatus* (Erichson, 1839)

Kemerovo Prov. (Babenko, 1982); Tomsk Prov. (Babenko, 1982).

**Genus** *Bolitochara* Mannerheim, 1830

*Bolitochara pulchra* (Gravenhorst, 1806)

Khanty-Mansi Distr. (Filippov, 2011); Tyumen Prov. (Bukhkalo et al., 2011, 2012); WS (Schülke, Smetana, 2015).

**Genus** *Encephalus* Stephens, 1832

*Encephalus complicans* Stephens, 1832

Tyumen Prov. (Bukhkalo et al., 2011, 2012); WS (Schülke, Smetana, 2015).

**Genus** *Gyrophaena* Mannerheim, 1830

*Gyrophaena affinis* Mannerheim, 1830

Kemerovo Prov. (Babenko, 1982); Tomsk Prov. (Babenko, 1982); Tyumen Prov. (Bukhkalo et al., 2011, 2012); WS (Schülke, Smetana, 2015).

*Gyrophaena bihamata* Thomson, 1867

Kurgan Prov. (Krasutsky, 2005); Sverdlovsk Prov. (Krasutsky, 2005); Tyumen Prov. (Bukhkalo et al., 2011, 2012); WS (Schülke, Smetana, 2015).

*Gyrophaena boleti* (Linnaeus, 1758)

Tyumen Prov. (Bukhkalo et al., 2011); WS (Schülke, Smetana, 2015).

*Gyrophaena congrua* Erichson, 1837

Tyumen Prov. (Bukhkalo et al., 2011, 2012); WS (Schülke, Smetana, 2015).

*Gyrophaena fasciata* (Marsham, 1802)

Tyumen Prov. (Bukhkalo et al., 2011, 2012).

*Gyrophaena gentilis* Erichson, 1839

Kemerovo Prov. (Babenko, 1982); Tomsk Prov. (Babenko, 1982); WS (Schülke, Smetana, 2015).

*Gyrophaena joyi* Wendeler, 1924

Tyumen Prov. (Bukhkalo et al., 2011); WS (Schülke, Smetana, 2015).

*Gyrophaena joyioides*Wüsthoff, 1937

Tyumen Prov. (Bukhkalo et al., 2011, 2012); WS (Schülke, Smetana, 2015).

*Gyrophaena manca* Erichson, 1839

Tyumen Prov. (Bukhkalo et al., 2011); WS (Schülke, Smetana, 2015).

*Gyrophaena nana* (Paykull, 1800)

Tyumen Prov. (Bukhkalo et al., 2011, 2012); WS (Schülke, Smetana, 2015).

*Gyrophaena nitidula* (Gyllenhal, 1810)

WS (Schülke, Smetana, 2015).

*Gyrophaena obsoleta* Ganglbauer, 1895

Tyumen Prov. (Bukhkalo et al., 2011).

*Gyrophaena orientalis* A.Strand, 1938

Tyumen Prov. (Bukhkalo et al., 2011); WS (Schülke, Smetana, 2015).

*Gyrophaena polita* (Gravenhorst, 1802)

WS (Schülke, Smetana, 2015).

*Gyrophaena poweri* Crotch, 1867

Tyumen Prov. (Bukhkalo et al., 2011, 2012); WS (Schülke, Smetana, 2015).

*Gyrophaena pseudonana* A.Strand, 1939

WS (Schülke, Smetana, 2015).

*Gyrophaena pulchella* Heer, 1839

Tyumen Prov. (Bukhkalo et al., 2011, 2012); WS (Schülke, Smetana, 2015).

*Gyrophaena rugipennis* Mulsant & Rey, 1861

WS (Schülke, Smetana, 2015).

*Gyrophaena strictula* Erichson, 1839

WS (Schülke, Smetana, 2015).

**Genus** *Homalota* Mannerheim, 1830

*Homalota nigricans* Thomson, 1871

WS (Schülke, Smetana, 2015).

*Homalota plana* (Gyllenhal, 1810)

Kemerovo Prov. (Babenko, 1982); Tomsk Prov. (Babenko, 1982, Kercheva et al., 2022); WS (Schülke, Smetana, 2015).

**Genus** *Leptusa* Kraatz, 1856

*Leptusa fumida* (Erichson, 1839)

WS (Schülke, Smetana, 2015).

**Genus** *Phymatura* J. Sahlberg, 1876

*Phymatura brevicollis* (Kraatz, 1856)

WS (Schülke, Smetana, 2015).

**Genus** *Rhopalocerina* Reitter, 1909

*Rhopalocerina clavigera* (W.Scriba, 1859)

Tyumen Prov. (Bukhkalo et al., 2011).

**Tribe** *Hygronomini* Thomson, 1859

**Genus** *Hygronoma* Erichson, 1837

*Hygronoma dimidiata* (Gravenhorst, 1806)

Tyumen Prov. (Bukhkalo et al., 2011, 2012); WS (Schülke, Smetana, 2015).

**Tribe** *Hypocyphtini* Laporte, 1835

**Genus** *Cypha* Leach, 1819

*Cypha laeviuscula* Mannerheim, 1830

Tyumen Prov. (Bukhkalo et al., 2011, 2012); WS (Schülke, Smetana, 2015).

**Genus** *Oligota* Mannerheim, 1830

*Oligota pusillima* (Gravenhorst, 1806)

WS (Schülke, Smetana, 2015).

**Tribe** *Lomechusini* Fleming, 1821

**Genus** *Drusilla* Leach, 1819

*Drusilla canaliculata* (Fabricius, 1787)

Kemerovo Prov. (Babenko et al., 1982, 2018); Khanty-Mansi Distr. (Filippov, 2011); Tomsk Prov. (Babenko et al., 1982, 2018; Babenko, Nuzhnykh, 2014); Tyumen Prov. (Bukhkalo et al., 2011, 2012); WS (Schülke, Smetana, 2015).

**Genus** *Lomechusa* Gravenhorst, 1806

*Lomechusa emarginata* (Paykull, 1789)

Tyumen Prov. (Bukhkalo et al., 2011, 2012).

*Lomechusa paradoxa* Gravenhorst, 1806

Kemerovo Prov. (Babenko, 1982); Tomsk Prov. (Babenko, 1982); Tyumen Prov. (Bukhkalo et al., 2011, 2012).

*Lomechusa pubicollis* Brisout de Barneville, 1860

Kemerovo Prov. (Babenko, 1982); Tomsk Prov. (Babenko, 1982); Tyumen Prov. (Bukhkalo et al., 2011, 2012).

**Genus** *Lomechusoides* Tottenham, 1939

*Lomechusoides sibiricus* (Motschulsky, 1844)

WS (Schülke, Smetana, 2015).

*Lomechusoides strumosus* (Fabricius, 1792)

Tyumen Prov. (Bukhkalo et al., 2011); WS (Schülke, Smetana, 2015).

**Genus** *Pella* Stephens, 1835

*Pella cognata* (Märkel, 1842)

Tyumen Prov. (Bukhkalo et al., 2011, 2012); WS (Schülke, Smetana, 2015).

*Pella humeralis* (Gravenhorst, 1802)

Tyumen Prov. (Bukhkalo et al., 2011, 2012); WS (Schülke, Smetana, 2015).

*Pella limbata* (Paykull, 1789)

Tyumen Prov. (Bukhkalo et al., 2011, 2012).

**Genus** *Zyras* Stephens, 1835

*Zyras collaris* Paykull, 1800

Tyumen Prov. (Bukhkalo et al., 2014).

**Tribe** *Myllaenini* Ganglbauer, 1895

**Genus** *Myllaena* Erichson, 1837

*Myllaena dubia* (Gravenhorst, 1806)

Tyumen Prov. (Bukhkalo et al., 2011, 2012); WS (Schülke, Smetana, 2015).

*Myllaena gracilis* (A.H. Matthews, 1838)

WS (Schülke, Smetana, 2015).

*Myllaena intermedia* Erichson, 1837

Tyumen Prov. (Bukhkalo et al., 2011, 2012).

*Myllaena minuta* (Gravenhorst, 1806)

Tyumen Prov. (Bukhkalo et al., 2011, 2012); WS (Schülke, Smetana, 2015).

**Tribe** *Oxypodini* Thomson, 1859

**Genus** *Calodera* Mannerheim, 1830

*Calodera aethiops* (Gravenhorst, 1802)

Tyumen Prov. (Bukhkalo et al., 2011, 2012); WS (Schülke, Smetana, 2015).

*Calodera nigrita* Mannerheim, 1830

WS (Schülke, Smetana, 2015).

**Genus** *Cephalocousya* Lohse, 1971

*Cephalocousya nivicola* (Thomson, 1871)

WS (Schülke, Smetana, 2015).

**Genus** *Devia* Blackwelder, 1952

*Devia prospera* (Erichson, 1839)

Tyumen Prov. (Bukhkalo et al., 2014); WS (Schülke, Smetana, 2015).

**Genus** *Hygropora* Kraatz, 1856

*Hygropora cunctans* (Erichson, 1837)

Tyumen Prov. (Bukhkalo et al., 2011, 2012); WS (Schülke, Smetana, 2015)

**Genus** *Ilyobates* Kraatz, 1856

*Ilyobates bennetti* Donisthorpe, 1914

WS (Schülke, Smetana, 2015).

**Genus** *Meotica* Mulsant & Rey, 1873

*Meotica exilis* (Knoch, 1806)

Tyumen Prov. (Bukhkalo et al., 2011, 2012); WS (Schülke, Smetana, 2015).

*Meotica filiformis* Motschulsky, 1860

Tyumen Prov. (Bukhkalo et al., 2011, 2012); WS (Schülke, Smetana, 2015).

**Genus** *Mniusa* Mulsant & Rey, 1875

*Mniusa grandiceps* (J.Sahlberg, 1876)

Tyumen Prov. (Bukhkalo et al., 2014); WS (Schülke, Smetana, 2015).

*Mniusa incrassata* (Mulsant & Rey, 1851)

Tyumen Prov. (Bukhkalo et al., 2011, 2012).

**Genus** *Pyroglossa* Bernhauer, 1901

*Neothetalia pulcherrima* (Bernhauer, 1901)

WS (Schülke, Smetana, 2015).

**Genus** *Ocalea* Erichson, 1837

*Ocalea badia* Erichson, 1837

WS (Schülke, Smetana, 2015).

**Genus** *Ocyusa* Kraatz, 1856

*Ocyusa maura* (Erichson, 1837)

Tyumen Prov. (Bukhkalo et al., 2011).

**Genus** *Oxypoda* Mannerheim, 1830

*Oxypoda abdominalis* (Mannerheim, 1830)

Tyumen Prov. (Bukhkalo et al., 2011, 2012); WS (Schülke, Smetana, 2015).

*Oxypoda acuminata* (Stephens, 1832)

WS (Schülke, Smetana, 2015).

*Oxypoda advena* Mäklin, 1846

Tyumen Prov. (Bukhkalo et al., 2011, 2012); WS (Schülke, Smetana, 2015).

*Oxypoda alternans* (Gravenhorst, 1802)

Tyumen Prov. (Bukhkalo et al., 2011, 2012).

*Oxypoda annularis* (Mannerheim, 1830)

Tyumen Prov. (Bukhkalo et al., 2011, 2012); WS (Schülke, Smetana, 2015).

*Oxypoda brachyptera* (Stephens, 1832)

Tyumen Prov. (Bukhkalo et al., 2011, 2012); WS (Schülke, Smetana, 2015).

*Oxypoda brevicornis* (Stephens, 1832)

Tyumen Prov. (Bukhkalo et al., 2011, 2012); WS (Schülke, Smetana, 2015).

*Oxypoda elongatula* Aube, 1850

Tyumen Prov. (Bukhkalo et al., 2011, 2012); WS (Schülke, Smetana, 2015).

*Oxypoda flavicornis* Kraatz, 1856

Tyumen Prov. (Bukhkalo et al., 2011, 2012); WS (Schülke, Smetana, 2015).

*Oxypoda funebris* Kraatz, 1856

Tyumen Prov. (Bukhkalo et al., 2011); WS (Schülke, Smetana, 2015).

*Oxypoda haemorrhoa* (Mannerheim, 1830)

Tyumen Prov. (Bukhkalo et al., 2011); WS (Schülke, Smetana, 2015).

*Oxypoda hansseni* A.Strand, 1946

Tyumen Prov. (Bukhkalo et al., 2011).

*Oxypoda islandica* Kraatz, 1857

Tyumen Prov. (Bukhkalo et al., 2011, 2012).

*Oxypoda lentula* Erichson, 1837

WS (Schülke, Smetana, 2015).

*Oxypoda longipes* Mulsant & Rey, 1861

Tyumen Prov. (Bukhkalo et al., 2011, 2012); WS (Schülke, Smetana, 2015).

*Oxypoda nigricornis* Motschulsky, 1860

WS (Schülke, Smetana, 2015).

*Oxypoda opaca* (Gravenhorst, 1802)

WS (Schülke, Smetana, 2015).

*Oxypoda praecox* Erichson, 1839

Tyumen Prov. (Bukhkalo et al., 2011, 2012); WS (Schülke, Smetana, 2015).

*Oxypoda procerula* Mannerheim, 1830

Tyumen Prov. (Bukhkalo et al., 2011, 2012); Yamalo-Nenets Distr. (Olshwang, 1992); WS (Schülke, Smetana, 2015).

*Oxypoda skalitzkyi* Bernhauer, 1902

Tyumen Prov. (Bukhkalo et al., 2011, 2012).

*Oxypoda soror* Thomson, 1855

Tyumen Prov. (Bukhkalo et al., 2011, 2012).

*Oxypoda togata* Erichson, 1837

Tyumen Prov. (Bukhkalo et al., 2011, 2012); WS (Schülke, Smetana, 2015).

**Genus** *Phloeopora* Erichson, 1837

*Phloeopora nitidiventris* Fauvel, 1900

Kemerovo Prov. (Babenko, 1982); Tomsk Prov. (Babenko, 1982).

*Phloeopora testacea* (Mannerheim, 1830)

Kemerovo Prov. (Babenko, 1982); Tomsk Prov. (Babenko, 1982).

**Genus** *Pyroglossa* Bernhauer, 1901

*Pyroglossa canaliculata* (Eppelsheim, 1893)

WS (Schülke, Smetana, 2015).

*Pyroglossa mirabilis* (Bernhauer, 1901)

WS (Schülke, Smetana, 2015).

*Pyroglossa opaca* Bernhauer, 1902

WS (Schülke, Smetana, 2015).

**Tribe** *Placusini* Mulsant & Rey, 1871

**Genus** *Placusa* Erichson, 1837

*Placusa atrata* (Mannerheim, 1830)

Kemerovo Prov. (Babenko, 1982); Tomsk Prov. (Babenko, 1982); Tyumen Prov. (Bukhkalo et al., 2011, 2012); WS (Schülke, Smetana, 2015).

*Placusa complanata* Erichson, 1839

Kemerovo Prov. (Babenko, 1982); Tomsk Prov. (Babenko, 1982, Kercheva, 2022).

*Placusa depressa* Mäklin, 1845

Kemerovo Prov. (Babenko, 1982); Tomsk Prov. (Babenko, 1982).

*Placusa pumilio* (Gravenhorst, 1802)

WS (Schülke, Smetana, 2015).

*Placusa tachyporoides* (Waltl, 1838)

Kemerovo Prov. (Babenko, 1982); Tomsk Prov. (Babenko, 1982); WS (Schülke, Smetana, 2015).

**Tribe** *Tachyusini* Thomson, 1859

**Genus** *Brachyusa* Mulsant & Rey, 1874

*Brachyusa concolor* (Erichson, 1839)

Tyumen Prov. (Bukhkalo et al., 2011, 2012).

**Genus** *Dasygnypeta* Lohse, 1974

*Dasygnypeta velata* (Erichson, 1837)

Tyumen Prov. (Bukhkalo et al., 2011, 2012); WS (Schülke, Smetana, 2015).

**Genus** *Dilacra* Thomson, 1858

*Dilacra luteipes* (Erichson, 1837)

Tyumen Prov. (Bukhkalo et al., 2011, 2012); WS (Schülke, Smetana, 2015).

*Dilacra vilis* (Erichson, 1837)

Tyumen Prov. (Bukhkalo et al., 2011, 2012).

**Genus** Gnypeta Thomson, 1858

*Gnypeta caerulea* (C.R. Sahlberg, 1830)

WS (Schülke, Smetana, 2015).

*Gnypeta canaliculata* J. Sahlberg, 1880

WS (Schülke, Smetana, 2015).

*Gnypeta carbonaria* (Mannerheim, 1830)

WS (Schülke, Smetana, 2015).

*Gnypeta cavicollis* J. Sahlberg, 1880

WS (Schülke, Smetana, 2015).

**Genus** *Ischnopoda* Stephens, 1835

*Ischnopoda leucopus* (Marsham, 1802)

Tyumen Prov. (Bukhkalo et al., 2011); WS (Schülke, Smetana, 2015).

*Ischnopoda umbratica* (Erichson, 1837)

WS (Schülke, Smetana, 2015).

**Genus** *Tachyusa* Erichson, 1837

*Tachyusa coarctata* Erichson, 1837

Tyumen Prov. (Bukhkalo et al., 2011, 2012); WS (Schülke, Smetana, 2015).

*Tachyusa concinna* Kraatz, 1856

WS (Schülke, Smetana, 2015).

*Tachyusa objecta* Mulsant & Rey, 1870

WS (Schülke, Smetana, 2015).

**Genus** *Thinonoma* Thomson, 1859

*Thinonoma atra* (Gravenhorst, 1806)

Tyumen Prov. (Bukhkalo et al., 2011).

**Subfamily** ***Euaesthetinae* Thomson, 1859**

**Tribe** *Euaesthetini* Thomson, 1859

**Genus** *Euaesthetus* Gravenhorst, 1806

*Euaesthetus bipunctatus* (Ljungh, 1804)

Novosibirsk Prov. (Pavlov, 2005); Tyumen Prov. (Bukhkalo et al., 2011, 2012); WS (Schülke, Smetana, 2015).

*Euaesthetus ruficapillus* (Lacordaire, 1835)

Tyumen Prov. (Bukhkalo et al., 2011, 2012); WS (Schülke, Smetana, 2015).

*Euaesthetus ruficollis* (Motschulsky, 1860)

Tyumen Prov. (Bukhkalo et al., 2011); Yamalo-Nenets Distr. (Olshwang, 1992).

*Euaesthetus superlatus* Peyerimhoff, 1937

Tyumen Prov. (Bukhkalo et al., 2014); WS (Schülke, Smetana, 2015).

**Subfamily *Micropeplinae* Leach, 1815**

**Genus** *Arrhenopeplus* Koch, 1937

*Arrhenopeplus tesserula* (Curtis, 1828)

WS (Schülke, Smetana, 2015).

**Genus** *Micropeplus* Latreille, 1809

*Micropeplus laevipennis* Eppelsheim, 1881

WS (Schülke, Smetana, 2015).

*Micropeplus porcatus* (Paykull, 1789)

Tyumen Prov. (Bukhkalo et al., 2011, 2012).

**Subfamily *Mycetoporinae*, Thomson, 1859**

**Tribe** *Mycetoporini* Thomson, 1859

**Genus** *Bolitobius* Leach, 1819

*Bolitobius castaneus* (Stephens, 1832)

Novosibirsk Prov. (Pavlov, 2005); Tyumen Prov. (Bukhkalo et al., 2011, 2012); WS (Schülke, Smetana, 2015).

*Bolitobius cingulatus* Mannerheim, 1830

Tyumen Prov. (Bukhkalo et al., 2011, 2012); WS (Schülke, Smetana, 2015).

**Genus** *Bryophacis* Reitter, 1909

*Bryophacis crassicornis* (Mäklin, 1847)

Tyumen Prov. (Bukhkalo et al., 2014).

*Bryophacis maklini* J.Sahlberg, 1871

WS (Schülke, Smetana, 2015).

*Bryophacis rufus* (Thomson, 1861)

WS (Schülke, Smetana, 2015).

*Bryoporus cernuus* (Gravenhorst, 1806)

Tyumen Prov. (Bukhkalo et al., 2011, 2012).

**Genus** *Carphacis* Gozis, 1886

*Carphacis striatus* (Olivier, 1795)

Novosibirsk Prov. (Pavlov, 2005); WS (Schülke, Smetana, 2015).

**Genus** *Ischnosoma* Stephens, 1829

*Ischnosoma bergrothi* (Hellen, 1925)

Tyumen Prov. (Bukhkalo et al., 2011, 2012); WS (Schülke, Smetana, 2015).

*Ischnosoma longicorne* (Mäklin, 1847)

Tyumen Prov. (Bukhkalo et al., 2011, 2012); WS (Schülke, Smetana, 2015).

*Ischnosoma splendidum* (Gravenhorst, 1806)

Kemerovo Prov. (Babenko, 1982); Tomsk Prov. (Babenko, 1982); Tyumen Prov. (Bukhkalo et al., 2011, 2012); WS (Schülke, Smetana, 2015).

**Genus** *Lordithon* Thomson, 1859

*Lordithon arcuatus* Solsky, 1871

Tyumen Prov. (Bukhkalo et al., 2011, 2012); WS (Schülke, Smetana, 2015).

*Lordithon bicolor* (Gravenhorst, 1806)

Khanty-Mansi Distr. (Krasutsky, 1997); Sverdlovsk Prov. (Krasutsky, 2005); Tyumen Prov. (Bukhkalo et al., 2011, 2012; Krasutsky, 2005).

*Lordithon lunulatus* (Linnaeus, 1760)

Kemerovo Prov. (Babenko, 1982); Kurgan Prov (Krasutsky, 2005); Khanty-Mansi Distr. (Filippov, 2011; Krasutsky, 1997, 2005); Novosibirsk Prov. (Pavlov, 2005); Tomsk Prov. (Babenko, 1982); Sverdlovsk Prov. (Krasutsky, 1995, 2005); Tyumen Prov. (Bukhkalo et al., 2011, 2012, Krasutsky, 2005);

*Lordithon pulchellus* (Mannerheim, 1830)

Kemerovo Prov. (Babenko, 1982); Novosibirsk Prov. (Pavlov, 2005); Tomsk Prov. (Babenko, 1982); Tyumen Prov. (Bukhkalo et al., 2011, 2012); WS (Schülke, Smetana, 2015).

*Lordithon speciosus* (Erichson, 1839)

Khanty-Mansi Distr. (Filippov, 2011); Tyumen Prov. (Bukhkalo et al., 2014); WS (Schülke, Smetana, 2015).

*Lordithon thoracicus* (Fabricius, 1777)

Kemerovo Prov. (Babenko, 1982); Khanty-Mansi Distr. (Filippov, 2011); Tomsk Prov. (Babenko, 1982); Tyumen Prov. (Bukhkalo et al., 2011, 2012); WS (Schülke, Smetana, 2015).

*Lordithon trimaculatus* (Fabricius, 1792)

Kemerovo Prov. (Babenko, 1982); Khanty-Mansi Distr. (Filippov, 2011; Krasutsky, 2005, 1997); Novosibirsk Prov. (Pavlov, 2005); Kurgan Prov. (Krasutsky, 2005); Sverdlovsk Prov. (Krasutsky, 2005); Tomsk Prov. (Babenko, 1982); Tyumen Prov. (Bukhkalo et al., 2011; Krasutsky, 2005); WS (Schülke, Smetana, 2015).

**Genus** *Mycetoporus* Mannerheim, 1830

*Mycetoporus additus* Eppelsheim, 1886

Tyumen Prov. (Bukhkalo et al., 2011, 2012); WS (Schülke, Smetana, 2015).

*Mycetoporus bimaculatus* Lacordaire, 1835

Tyumen Prov. (Bukhkalo et al., 2011, 2012); WS (Schülke, Smetana, 2015).

*Mycetoporus clavicornis* (Stephens, 1832)

Tyumen Prov. (Bukhkalo et al., 2011, 2012).

*Mycetoporus glaber* (Sperk, 1835)

Tyumen Prov. (Bukhkalo et al., 2011, 2012); WS (Schülke, Smetana, 2015).

*Mycetoporus lepidus* (Gravenhorst, 1806)

Khanty-Mansi Distr. (Krasutsky, 1997, 2005); Tyumen Prov. (Bukhkalo et al., 2011, 2012); WS (Schülke, Smetana, 2015).

*Mycetoporus longulus* Mannerheim, 1830

Tyumen Prov. (Bukhkalo et al., 2011, 2012); WS (Schülke, Smetana, 2015).

*Mycetoporus maerkelii* Kraatz, 1857

Tyumen Prov. (Bukhkalo et al., 2011, 2012).

*Mycetoporus montanus* Luze, 1901

Tyumen Prov. (Bukhkalo et al., 2011, 2012).

*Mycetoporus mulsanti* (Ganglbauer, 1895)

Tyumen Prov. (Bukhkalo et al., 2014).

*Mycetoporus nigrans* Mäklin, 1853

WS (Schülke, Smetana, 2015).

*Mycetoporus pachyraphis* (Pandellé, 1869)

Tyumen Prov. (Bukhkalo et al., 2011, 2012).

*Mycetoporus punctus* (Gravenhorst, 1806)

WS (Schülke, Smetana, 2015).

**Subfamily *Olisthaerinae* Thompson, 1858**

**Genus** *Olisthaerus* Dejean, 1833

*Olisthaerus megacephalus* (Zetterstedt, 1828)

WS (Schülke, Smetana, 2015).

*Olisthaerus substriatus*(Paykull, 1790)

Kemerovo Prov. (Babenko, 1982); Tomsk Prov. (Babenko, 1982); WS (Schülke, Smetana, 2015).

**Subfamily *Omaliinae MacLeay, 1825***

**Tribe** *Anthophagini* Thomson, 1859

**Genus** *Acidota* Stephens, 1829

*Acidota crenata*(Fabricius, 1792)

Kemerovo Prov. (Babenko, 1982); Khanty-Mansi Distr. (Shavrin, 2021); Tomsk Prov. (Babenko, 1982; Babenko, Nuzhnykh, 2014); Tyumen Prov. (Bukhkalo et al., 2011, 2012; Shavrin, 2021); Yamalo-Nenets Distr. (Shavrin, 2021); WS (Schülke, Smetana, 2015).

*Acidota cruentata*Mannerheim, 1830

Kemerovo Prov. (Babenko, 1982); Khanty-Mansi Distr. (Zinovyev et al., 2016); Novosibirsk Prov. (Shavrin, 2021); Tomsk Prov. (Babenko, 1982); Tyumen Prov. (Bukhkalo et al., 2011, 2012); WS (Schülke, Smetana, 2015).

*Acidota quadrata*(Zetterstedt, 1838)

Khanty-Mansi Distr. (Shavrin, 2021); Yamalo-Nenets Distr. (Shavrin, 2021); WS (Schülke, Smetana, 2015).

*Anthobium* Leach, 1819

*Anthobium atrocephalum*(Gyllenhal, 1827)

WS (Schülke, Smetana, 2015).

*Anthobium melanocephalum*(Illiger, 1794)

Kurgan Prov. (Krasutsky, 2005); Sverdlovsk Prov (Krasutsky, 2005).

**Genus** *Anthophagus* Gravenhorst*, 1802*

*Anthophagus angusticollis* (Mannerheim, 1830)

WS (Schülke, Smetana, 2015).

*Anthophagus caraboides*(Linnaeus, 1758)

Khanty-Mansi Distr. (Filippov, 2011); Novosibirsk Prov. (Pavlov, 2005); Tyumen Prov. (Bukhkalo et al., 2011, 2012); WS (Schülke, Smetana, 2015).

*Anthophagus omalinus*Zetterstedt, 1828

Khanty-Mansi Distr. (Filippov, 2011); Tyumen Prov. (Bukhkalo et al., 2011, 2012); WS (Schülke, Smetana, 2015).

*Arpedium* Erichson, 1839

*Arpedium brachypterum*(Gravenhorst, 1802)

Khanty-Mansi Distr. (Filippov, 2011); Tyumen Prov. (Bukhkalo et al., 2011, 2012); WS (Schülke, Smetana, 2015).

*Arpedium brunnescens*J.Sahlberg, 1871

WS (Schülke, Smetana, 2015); Yamalo-Nenets Distr. (Olshvang, 1992).

*Arpedium puncticolle*J.Sahlberg, 1880

Khanty-Mansi Distr. (Filippov, 2011); Tyumen Prov. (Bukhkalo et al., 2011, 2012); WS (Schülke, Smetana, 2015).

*Arpedium quadrum*(Gravenhorst, 1806)

Kemerovo Prov. (Babenko, 1982); Khanty-Mansi Distr. (Filippov, 2011); Novosibirsk Prov. (Pavlov, 2005); Tomsk Prov. (Babenko, 1982); Tyumen Prov. (Bukhkalo et al., 2011, 2012); WS (Schülke, Smetana, 2015).

*Arpedium tenue*(LeConte, 1863)

WS (Schülke, Smetana, 2015).

**Genus** *Cylletron* Thomson, 1859

*Cylletron nivale*Thomson, 1859

WS (Schülke, Smetana, 2015).

**Genus** *Deliphrum* Erichson, 1839

*Deliphrum tectum*(Paykull, 1789)

Kemerovo Prov. (Babenko, 1982); Tomsk Prov. (Babenko, 1982); Tyumen Prov. (Bukhkalo et al., 2014); WS (Schülke, Smetana, 2015).

**Genus** *Geodromicus* L. Redtenbacher, 1857

*Geodromicus plagiatus (Fabricius, 1798)*

Kemerovo Prov. (Babenko, 1982); Tomsk Prov. (Babenko, 1982); WS (Schülke, Smetana, 2015).

**Genus** *Mannerheimia* Mäklin, 1880

*Mannerheimia arctica*(Erichson, 1840)

Khanty-Mansi Distr. (Shavrin, 2021); Tyumen Prov. (Shavrin, 2021); Yamalo-Nenets Distr. (Shavrin, 2021); WS (Schülke, Smetana, 2015).

*Mannerheimia brevipennis*(Motschulsky, 1860)

Khanty-Mansi Distr. (Shavrin, 2021); Yamalo-Nenets Distr. (Shavrin, 2021); WS (Schülke, Smetana, 2015).

*Olophrum* Erichson, 1839

*Olophrum boreale*(Paykull, 1792)

Khanty-Mansi Distr. (Filippov, 2011); Yamalo-Nenets Distr. (Olshvang, 1992); WS (Schülke, Smetana, 2015).

*Olophrum consimile*(Gyllenhal, 1810)

Khanty-Mansi Distr. (Filippov, 2011); Tomsk Prov. (Babenko, Nuzhnykh, 2014); Tyumen Prov. (Bukhkalo et al., 2011, 2012).

*Olophrum fuscum*(Gravenhorst, 1806)

WS (Schülke, Smetana, 2015).

*Olophrum latum*Mäklin, 1853

WS (Schülke, Smetana, 2015).

*Olophrum rotundicolle*(C.R.Sahlberg, 1830)

Khanty-Mansi Distr. (Zinovyev et al., 2016); Yamalo-Nenets Distr. (Olshvang, 1992); WS (Schülke, Smetana, 2015).

*Porrhodites* Kraatz, 1857

*Porrhodites fenestralis*(Zetterstedt, 1828)

WS (Schülke, Smetana, 2015).

**Tribe** *Coryphiini* Jakobson, 1908

**Genus** *Boreaphilus* C.R. Sahlberg, 1832

*Boreaphilus henningianus*C.R.Sahlberg, 1832

WS (Schülke, Smetana, 2015).

**Genus** *Eudectus* L. Redtenbacher, 1857

*Eudectus whitei*Sharp, 1871

Khanty-Mansi Distr (Filippov, 2011).

**Genus** *Holoboreaphilus* Campbell, 1978

*Holoboreaphilus nordenskioldi* Mäklin, 1878

WS (Schülke, Smetana, 2015).

**Tribe** *Eusphalerini* Hatch, 1957

**Genus** *Eusphalerum* Kraatz, 1857

*Eusphalerum luteum*(Marsham, 1802)

Tyumen Prov. (Bukhkalo et al., 2011, 2012); WS (Schülke, Smetana, 2015).

*Eusphalerum minutum*(Fabricius, 1792)

Kemerovo Prov. (Babenko, 1982); Tomsk Prov. (Babenko, 1982); Tyumen Prov. (Bukhkalo et al., 2014); WS (Schülke, Smetana, 2015).

*Eusphalerum sibiricum*(Luze, 1910)

WS (Schülke, Smetana, 2015).

*Eusphalerum subsolanum*Herman, 2001

WS (Schülke, Smetana, 2015).

*Eusphalerum tenenbaumi*(Bernhauer, 1932)

Kemerovo Prov. (Babenko, 1982); Tomsk Prov. (Babenko, 1982); WS (Schülke, Smetana, 2015).

**Tribe** *Omaliini* McLeay, 1825

**Genus** *Acrolocha* Thomson, 1858

*Acrolocha pliginskii*Bernhauer, 1912

WS (Schülke, Smetana, 2015).

**Genus** *Acrulia Thomson*, 1858

*Acrulia inflata*(Gyllenhal, 1813)

Khanty-Mansi Distr. (Krasutsky, 1997, 2005); Kurgan Prov. (Krasutsky, 2005); Sverdlovsk Prov (Krasutsky, 2005); Tyumen Prov. (Krasutsky, 2005); WS (Schülke, Smetana, 2015).

**Genus** *Dropephylla* Mulsant & Rey, 1880

*Dropephylla* *puella* Tanabe & Nakane, 1990

WS (Schülke, Smetana, 2015).

**Genus** *Micralymma* Westwood, 1838

*Micralymma brevilingue*Schiodte, 1845

Khanty-Mansi Distr. (Zinovyev et al., 2016); WS (Schülke, Smetana, 2015).

**Genus** *Omalium* Gravenhorst, 1802

*Omalium caesum*Gravenhorst, 1806

Tyumen Prov. (Bukhkalo et al., 2011, 2012); WS (Schülke, Smetana, 2015).

*Omalium curtipenne*(Mäklin, 1878)

WS (Schülke, Smetana, 2015).

*Omalium excavatum*Stephens, 1834

Kemerovo Prov. (Babenko, 1982); Tomsk Prov. (Babenko, 1982); Tyumen Prov. (Bukhkalo et al., 2014).

*Omalium oxyacanthae*Gravenhorst, 1806

WS (Schülke, Smetana, 2015).

*Omalium rivulare*(Paykull, 1789)

Tyumen Prov. (Bukhkalo et al., 2011, 2012).

*Omalium strigicolle*Wankowicz, 1869

WS (Schülke, Smetana, 2015).

**Genus** *Phloeonomus* Heer, 1839

*Phloeonomus pusillus* (Gravenhorst, 1806)

Kemerovo Prov. (Babenko, 1982); Tomsk Prov. (Babenko, 1982); WS (Schülke, Smetana, 2015).

*Phloeonomus sjobergi*A.Strand, 1937

Tomsk Prov. (Kercheva et al., 2022).

**Genus** *Phloeostiba* Thomson, 1858

*Phloeostiba lapponica*(Zetterstedt, 1838)

Kemerovo Prov. (Babenko, 1982); Tomsk Prov. (Babenko, 1982); Tyumen Prov. (Bukhkalo et al., 2011, 2012); WS (Schülke, Smetana, 2015).

*Phloeostiba plana*(Paykull, 1792)

Tyumen Prov. (Bukhkalo et al., 2011, 2012); WS (Schülke, Smetana, 2015).

**Genus** *Phyllodrepa* Thomson, 1859

*Phyllodrepa nigra*(Gravenhorst, 1806)

WS (Schülke, Smetana, 2015).

*Phyllodrepa rufipennis*Luze, 1906

WS (Schülke, Smetana, 2015).

**Genus** *Pycnoglypta Thomson*, 1858

*Pycnoglypta heydeni*Eppelsheim, 1886

WS (Schülke, Smetana, 2015).

*Pycnoglypta lurida*(Gyllenhal, 1813)

WS (Schülke, Smetana, 2015).

**Genus** *Xylodromus* Heer, 1839

*Xylodromus depressus*(Gravenhorst, 1802)

Kemerovo Prov. (Babenko, 1982); Tomsk Prov. (Babenko, 1982); WS (Schülke, Smetana, 2015).

**Subfamily *Oxyporinae* Fleming, 1821**

**Genus** *Oxyporus* Fabricius, 1775

*Oxyporus* *aequicollis* Bernhauer, 1935

Kemerovo Prov. (Babenko, 1982); Tomsk Prov. (Babenko, 1982).

*Oxyporus mannerheimii*Gyllenhal, 1827

Kemerovo Prov. (Babenko, 1982); Khanty-Mansi Distr. (Krasutsky, 1997, 2005); Kurgan Prov. (Krasutsky, 2005); Sverdlovsk Prov. (Krasutsky, 1995, 2005); Tomsk Prov. (Babenko, 1982); Tyumen Prov. (Bukhkalo et al., 2011, Krasutsky, 2005); WS (Schülke, Smetana, 2015).

*Oxyporus maxillosus*Fabricius, 1792

Khanty-Mansi Distr. (Krasutsky, 1997, 2005); Kurgan Prov. (Krasutsky, 2005); Novosibirsk Prov. (Pavlov, 2005); Sverdlovsk Prov. (Krasutsky, 1995, 2005);

Tomsk Prov. (Babenko, 1982); Tyumen Prov. (Bukhkalo et al., 2011, 2012; Krasutsky, 2005); WS (Schülke, Smetana, 2015).

*Oxyporus rufus*(Linnaeus, 1758)

Kemerovo Prov. (Babenko, 1982); Khanty-Mansi Distr. (Krasutsky, 2005); Kurgan Prov. (Krasutsky, 2005); Novosibirsk Prov. (Pavlov, 2005); Sverdlovsk Prov (Krasutsky, 2005);

Tomsk Prov. (Babenko, 1982); Tyumen Prov. (Bukhkalo et al., 2011, 2012); WS (Schülke, Smetana, 2015).

**Subfamily *Oxytelinae* Fleming, 1821**

**Tribe** *Blediini* Adam, 2001

**Genus** *Bledius* Leach, 1819

*Bledius bicornis*(Germar, 1822)

Novosibirsk Prov. (Pavlov, 2005); WS (Schülke, Smetana, 2015).

*Bledius denticollis*Fauvel, 1872

WS (Schülke, Smetana, 2015).

*Bledius dissimilis*Erichson, 1840

Novosibirsk Prov. (Pavlov, 2005); Tyumen Prov. (Bukhkalo et al., 2011, 2012); WS (Schülke, Smetana, 2015).

*Bledius erraticus*Erichson, 1839

Novosibirsk Prov. (Pavlov, 2005); WS (Schülke, Smetana, 2015).

*Bledius fergussoni* Joy, 1912

WS (Schülke, Smetana, 2015).

*Bledius gallicus*(Gravenhorst, 1806)

Novosibirsk Prov. (Pavlov, 2005); Tyumen Prov. (Bukhkalo et al., 2011, 2012); WS (Schülke, Smetana, 2015).

*Bledius littoralis*Heer, 1839

Kemerovo Prov. (Babenko, 1982); Novosibirsk Prov. (Pavlov, 2005); Tomsk Prov. (Babenko, 1982); WS (Schülke, Smetana, 2015).

*Bledius opacus*(Block, 1799)

Tyumen Prov. (Bukhkalo et al., 2011, 2012); WS (Schülke, Smetana, 2015).

*Bledius pallipes*(Gravenhorst, 1806)

Tyumen Prov. (Bukhkalo et al., 2011, 2012); WS (Schülke, Smetana, 2015).

*Bledius poppiusi*Bernhauer, 1902

WS (Schülke, Smetana, 2015).

*Bledius procerulus*Erichson, 1840

Novosibirsk Prov. (Pavlov, 2005); Tyumen Prov. (Bukhkalo et al., 2011, 2012); WS (Schülke, Smetana, 2015).

*Bledius rugosulus*Eppelsheim, 1893

WS (Schülke, Smetana, 2015).

*Bledius spectabilis Kraatz,* 1857

Novosibirsk Prov. (Pavlov, 2005); WS (Schülke, Smetana, 2015).

*Bledius subterraneus*Erichson, 1839

WS (Schülke, Smetana, 2015).

*Bledius tenenbaumi*Bernhauer, 1936

Tyumen Prov. (Bukhkalo et al., 2011, 2012); WS (Schülke, Smetana, 2015).

*Bledius tricornis*(Herbst, 1784)

Novosibirsk Prov. (Pavlov, 2005); WS (Schülke, Smetana, 2015).

**Tribe** *Coprophilini* Heer, 1839

**Genus** *Coprophilus* Latreille, 1829

*Coprophilus schubertii*(Motschulsky, 1860)

Kemerovo Prov. (Zinchenko, 2003).

**Tribe** *Deleasteriini* Reitter, 1909

**Genus** *Deleaster* Erichson, 1839

*Deleaster dichrous*(Gravenhorst, 1802)

Kemerovo Prov. (Babenko, 1982); Tomsk Prov. (Babenko, 1982); Tyumen Prov. (Bukhkalo et al., 2011, 2012); WS (Schülke, Smetana, 2015).

**Tribe** *Oxytelini* Fleming, 1821

**Genus** *Anotylus* Thomson, 1859

*Anotylus insecatus*(Gravenhorst, 1806)

Kemerovo Prov. (Babenko, 1982); Tomsk Prov. (Babenko, 1982); Tyumen Prov. (Bukhkalo et al., 2011, 2012).

*Anotylus inustus*(Gravenhorst, 1806)

Novosibirsk Prov. (Pavlov, 2005).

*Anotylus nitidulus*(Gravenhorst, 1802)

Kemerovo Prov. (Babenko, 1982); Novosibirsk Prov. (Pavlov, 2005); Tomsk Prov. (Babenko, 1982); WS (Schülke, Smetana, 2015).

*Anotylus rugosoides*Schülke, 2012

WS (Schülke, Smetana, 2015).

*Anotylus rugosus*(Fabricius, 1775)

Kemerovo Prov. (Babenko, 1982); Novosibirsk Prov. (Pavlov, 2005); Tomsk Prov. (Babenko, 1982; Babenko, Nuzhnykh, 2014); Tyumen Prov. (Bukhkalo et al., 2011, 2012); WS (Schülke, Smetana, 2015).

*Anotylus sculpturatus*(Gravenhorst, 1806)

Kemerovo Prov. (Babenko, 1982); Tomsk Prov. (Babenko, 1982).

*Anotylus tetracarinatus*(Block, 1799)

Kemerovo Prov. (Babenko, 1982); Tomsk Prov. (Babenko, 1982).

**Genus** *Oxytelus* Gravenhorst, 1802

*Oxytelus laqueatus*(Marsham, 1802)

Kemerovo Prov. (Babenko, 1982); Tomsk Prov. (Babenko, 1982); WS (Schülke, Smetana, 2015).

*Oxytelus piceus*(Linnaeus, 1767)

Kemerovo Prov. (Babenko, 1982); Novosibirsk Prov. (Pavlov, 2005); Tomsk Prov. (Babenko, 1982); WS (Schülke, Smetana, 2015).

*Oxytelus sculptus*Gravenhorst, 1806

Kemerovo Prov. (Babenko, 1982); Tomsk Prov. (Babenko, 1982).

**Genus** *Platystethus* Mannerheim, 1830

*Platystethus arenarius*(Geoffroy, 1785)

Kemerovo Prov. (Babenko, 1982; Zinchenko, 2003); Novosibirsk Prov. (Pavlov, 2005); Tomsk Prov. (Babenko, 1982); Tyumen Prov. (Bukhkalo et al., 2011, 2012); WS (Schülke, Smetana, 2015).

*Platystethus capito*Heer, 1839

Kemerovo Prov. (Babenko, 1982); Novosibirsk Prov. (Pavlov, 2005); Tomsk Prov. (Babenko, 1982); Tyumen Prov. (Bukhkalo et al., 2011, 2012); WS (Schülke, Smetana, 2015).

*Platystethus cornutus* Gravenhorst, 1802

WS (Schülke, Smetana, 2015).

*Platystethus nitens*(C.R.Sahlberg, 1832)

Novosibirsk Prov. (Pavlov, 2005); Tyumen Prov. (Bukhkalo et al., 2011, 2012); WS (Schülke, Smetana, 2015).

*Platystethus nodifrons*Mannerheim, 1830

Tyumen Prov. (Bukhkalo et al., 2011, 2012); WS (Schülke, Smetana, 2015).

**Tribe** *Planeustomini* Jacquelin du Val, 1857

**Genus** *Manda* Blackwelder, 1952

*Manda mandibularis* (Gyllenhal, 1827)

Tyumen Prov. (Bukhkalo et al., 2011, 2012); WS (Schülke, Smetana, 2015).

**Tribe** *Thinobiini* J. Sahlberg, 1876

**Genus** *Aploderus* Stephens, 1833

*Aploderus caelatus*(Gravenhorst, 1802)

Kemerovo Prov. (Babenko, 1982); Tomsk Prov. (Babenko, 1982; Babenko, Nuzhnykh, 2014); WS (Schülke, Smetana, 2015).

**Genus** *Carpelimus* Leach, 1819

*Carpelimus bilineatus*(Stephens, 1834)

Novosibirsk Prov. (Pavlov, 2005); WS (Schülke, Smetana, 2015).

*Carpelimus corticinus*(Gravenhorst, 1806)

Tyumen Prov. (Bukhkalo et al., 2011, 2012).

*Carpelimus despectus*(Baudi di Selve, 1870)

Tyumen Prov. (Bukhkalo et al., 2011, 2012); WS (Schülke, Smetana, 2015).

*Carpelimus elongatulus*(Erichson, 1839)

Tyumen Prov. (Bukhkalo et al., 2011, 2012).

*Carpelimus exiguus*(Erichson, 1839)

Tyumen Prov. (Bukhkalo et al., 2011, 2012); WS (Schülke, Smetana, 2015).

*Carpelimus foveolatus*(C.R.Sahlberg, 1832)

Novosibirsk Prov. (Pavlov, 2005); WS (Schülke, Smetana, 2015).

*Carpelimus gracilis*(Mannerheim, 1830)

Tyumen Prov. (Bukhkalo et al., 2011, 2012); WS (Schülke, Smetana, 2015).

*Carpelimus impressus*(Lacordaire, 1835)

WS (Schülke, Smetana, 2015).

*Carpelimus lindrothi*(Palm, 1943)

Tyumen Prov. (Bukhkalo et al., 2011, 2012).

*Carpelimus manchuricus*(Bernhauer, 1938)

WS (Schülke, Smetana, 2015).

*Carpelimus modestus*(Casey, 1889)

Tyumen Prov. (Bukhkalo et al., 2011, 2012).

*Carpelimus obesus*(Kiesenwetter, 1844)

Tyumen Prov. (Bukhkalo et al., 2011, 2012).

*Carpelimus poppiusi*(Bernhauer & K.Schubert, 1911)

WS (Schülke, Smetana, 2015).

*Carpelimus pusillus*(Gravenhorst, 1802)

Tyumen Prov. (Bukhkalo et al., 2011, 2012); WS (Schülke, Smetana, 2015).

*Carpelimus rivularis*(Motschulsky, 1860)

Tyumen Prov. (Bukhkalo et al., 2011, 2012); WS (Schülke, Smetana, 2015).

*Carpelimus subtilis*(Erichson, 1839)

Tyumen Prov. (Bukhkalo et al., 2011, 2012); WS (Schülke, Smetana, 2015).

**Genus** *Thinodromus* Kraatz, 1857

*Thinodromus arcuatus*(Stephens, 1834)

Kemerovo Prov. (Babenko, 1982); Tomsk Prov. (Babenko, 1982); WS (Schülke, Smetana, 2015).

**Subfamily *Paederinae* Fleming, 1821**

**Tribe** *Paederini* Fleming, 1821

**Genus** *Astenus* Dejean, 1833

*Astenus gracilis* (Paykull, 1789)

Kemerovo Prov. (Babenko, 1982); Tomsk Prov. (Babenko, 1982); Tyumen Prov. (Bukhkalo et al., 2011, 2012); WS (Schülke, Smetana, 2015).

*Astenus procerus* (Gravenhorst, 1806)

Novosibirsk Prov. (Pavlov, 2005); WS (Schülke, Smetana, 2015).

**Genus** *Achenium* Leach, 1819

*Achenium humile* (Nicolai, 1822)

Novosibirsk Prov. (Pavlov, 2005); WS (Schülke, Smetana, 2015).

**Genus** *Lathrobium* Gravenhorst, 1802

*Lathrobium brunnipes* (Fabricius, 1792)

Kemerovo Prov. (Babenko, 1982; Ryvkin, 2011); Khanty-Mansi Distr. (Filippov, 2011; Ryvkin, 2011); Novosibirsk Prov. (Pavlov, 2005); Tomsk Prov. (Babenko, 1982, 2014); Tyumen Prov. (Bukhkalo et al., 2011, 2012; Ryvkin, 2021); WS (Schülke, Smetana, 2015).

*Lathrobium dimidiatipenne* Bernhauer, 1910

WS (Schülke, Smetana, 2015).

*Lathrobium elongatum* (Linnaeus, 1767)

Kemerovo Prov. (Babenko, 1982); Khanty-Mansi Distr. (Ryvkin, 2011); Novosibirsk Prov. (Pavlov, 2005); Tomsk Prov. (Babenko, 1982); Tyumen Prov. (Bukhkalo et al., 2011, 2012); WS (Schülke, Smetana, 2015).

*Lathrobium flavipes* Hochhuth, 1851

WS (Schülke, Smetana, 2015).

*Lathrobium fovulum* Stephens, 1833

Khanty-Mansi Distr. (Ryvkin, 2011); Novosibirsk Prov. (Pavlov, 2005); Tomsk Prov. (Babenko, 1982); Tyumen Prov. (Bukhkalo et al., 2011, 2012); WS (Schülke, Smetana, 2015).

*Lathrobium geminum* Kraatz, 1857

Kemerovo Prov. (Babenko, 1982); Khanty-Mansi Distr. (Ryvkin, 2011); Novosibirsk Prov. (Pavlov, 2005); Tomsk Prov. (Babenko, 1982); Tyumen Prov. (Bukhkalo et al., 2011, 2012; Ryvkin, 2011); WS (Schülke, Smetana, 2015).

*Lathrobium impressum* Heer, 1841

Novosibirsk Prov. (Pavlov, 2005); Tyumen Prov. (Bukhkalo et al., 2011, 2012; Ryvkin, 2011); WS (Schülke, Smetana, 2015).

*Lathrobium latum* Tikhomirova, 1968

Kemerovo Prov. (Babenko, 1982); Tomsk Prov. (Babenko, 1982); WS (Schülke, Smetana, 2015).

*Lathrobium longulum* Gravenhorst, 1802

Tyumen Prov. (Bukhkalo et al., 2011, 2012; Ryvkin, 2011); WS (Schülke, Smetana, 2015).

*Lathrobium poljarne* Coiffait, 1972

Yamalo-Nenets Distr. (Ryvkin, 2007); WS (Schülke, Smetana, 2015).

*Lathrobium rufipenne* Gyllenhal, 1813

Kemerovo Prov. (Babenko, 1982); Khanty-Mansi Distr. (Ryvkin, 2011); Tomsk Prov. (Babenko, 1982); WS (Schülke, Smetana, 2015).

**Genus** *Lobrathium* Mulsant & Rey, 1878

*Lobrathium indubium* (Eppelsheim, 1893)

WS (Schülke, Smetana, 2015).

**Genus** *Platydomene* Ganglbauer, 1895

*Platydomene picipes* (Erichson, 1840)

Novosibirsk Prov. (Pavlov, 2005).

**Genus** *Tetartopeus* Czwalina, 1888

*Tetartopeus baicalicus* (Eppelsheim, 1878)

WS (Schülke, Smetana, 2015).

*Tetartopeus quadratus* (Paykull, 1789)

Kemerovo Prov. (Babenko, 1982); Tomsk Prov. (Babenko, 1982); Tyumen Prov. (Bukhkalo et al., 2011, 2012); WS (Schülke, Smetana, 2015).

*Tetartopeus terminatus* (Gravenhorst, 1802)

Novosibirsk Prov. (Pavlov, 2005); Tyumen Prov. (Bukhkalo et al., 2011, 2012); WS (Schülke, Smetana, 2015).

*Tetartopeus zetterstedti* (Rye, 1872)

WS (Schülke, Smetana, 2015).

**Genus** *Sunius* Stephens, 1829

*Sunius propinquus* (Brisout de Barneville, 1867)

Novosibirsk Prov. (Pavlov, 2005).

**Genus** *Scopaeus* Erichson, 1839

*Scopaeus gracilis* (Sperk, 1835)

Novosibirsk Prov. (Pavlov, 2005).

*Scopaeus laevigatus* (Gyllenhal, 1827)

Tyumen Prov. (Bukhkalo et al., 2011, 2012); WS (Schülke, Smetana, 2015).

*Scopaeus pusillus* Kiesenwetter, 1843

WS (Schülke, Smetana, 2015).

**Genus** *Rugilus* Leach, 1819

*Rugilus angustatus* (Geoffroy, 1785)

Novosibirsk Prov. (Pavlov, 2005); WS (Schülke, Smetana, 2015).

*Rugilus geniculatus* (Erichson, 1839)

Novosibirsk Prov. (Pavlov, 2005).

*Rugilus rufipes* Germar, 1836

Novosibirsk Prov. (Pavlov, 2005); Tyumen Prov. (Bukhkalo et al., 2011, 2012).

*Rugilus similis* (Erichson, 1839*)*

Tyumen Prov. (Bukhkalo et al., 2011, 2012); WS (Schülke, Smetana, 2015).

**Tribe** *Paederini* Fleming, 1821

**Genus** *Leptobium* Casey, 1905

*Leptobium gracile* (Gravenhorst, 1802)

Novosibirsk Prov. (Pavlov, 2005).

**Genus** *Ochthephilum* Stephens, 1829

*Ochthephilum fracticorne* (Paykull, 1800)

Kemerovo Prov. (Babenko, 1982); Novosibirsk Prov. (Pavlov, 2005); Tomsk Prov. (Babenko, 1982); Tyumen Prov. (Bukhkalo et al., 2011, 2012); WS (Schülke, Smetana, 2015).

**Genus** *Paederus* Fabricius, 1775

*Paederus fuscipes* Curtis, 1826

Kemerovo Prov. (Babenko, 1982); Tomsk Prov. (Babenko, 1982); WS (Schülke, Smetana, 2015).

*Paederus littoralis* Bernhauer, 1932

Kemerovo Prov. (Babenko, 1982); Tomsk Prov. (Babenko, 1982); WS (Schülke, Smetana, 2015).

*Paederus riparius* (Linnaeus, 1758)

Kemerovo Prov. (Babenko, 1982); Khanty-Mansi Distr. (Filippov, 2011; Zinovyev, Akopyan, 2013); Novosibirsk Prov. (Pavlov, 2005); Tomsk Prov. (Babenko, 1982); Tyumen Prov. (Bukhkalo et al., 2011, 2012); WS (Schülke, Smetana, 2015).

**Subfamily** ***Proteininae* Erichson, 1839**

**Tribe** *Proteinini* Erichson, 1839

**Genus** *Megarthrus* Stephens, 1829

*Megarthrus denticollis* (Beck, 1817)

Kurgan Prov. (Krasutsky, 2005); Sverdlovsk Prov (Krasutsky, 2005); WS (Schülke, Smetana, 2015).

*Megarthrus depressus* (Paykull, 1789)

Kemerovo Prov. (Babenko, 1982); Tomsk Prov. (Babenko, 1982); Sverdlovsk Prov (Krasutsky, 2005); Tyumen Prov. (Bukhkalo et al., 2011, 2012, 2005); WS (Schülke, Smetana, 2015).

*Megarthrus hemipterus* (Illiger, 1794)

Kemerovo Prov. (Babenko, 1982); Khanty-Mansi Distr. (Krasutsky, 1997, 2005); Kurgan Prov. (Krasutsky, 2005); Sverdlovsk Prov (Krasutsky, 2005); Tomsk Prov. (Babenko, 1982; Babenko, Nuzhnykh, 2014); Tyumen Prov. (Krasutsky, 2005).

*Megarthrus nitidulus* Kraatz, 1857

WS (Schülke, Smetana, 2015).

*Megarthrus prosseni* Schatzmayr, 1904

WS (Schülke, Smetana, 2015).

**Genus** *Metopsia* Wollaston, 1854

*Metopsia clypeata* (P. Müller, 1821)

Novosibirsk Prov. (Pavlov, 2005).

**Genus** *Proteinus* Latreille, 1797

*Proteinus altaicus* Reitter, 1905

WS (Schülke, Smetana, 2015).

*Proteinus brachypterus* (Fabricius, 1792)

Tyumen Prov. (Bukhkalo et al., 2011, 2012); WS (Schülke, Smetana, 2015).

*Proteinus laevigatus* Hochhuth, 1872

Tyumen Prov. (Bukhkalo et al., 2011, 2012); WS (Schülke, Smetana, 2015).

**Subfamily *Pselaphinae* Latreille, 1802**

**Tribe** *Brachyglutini* Raffray, 1904

**Genus** *Brachygluta* Thomson, 1859

*Brachygluta fossulata* (Reichenbach, 1816)

Tyumen Prov. (Bukhkalo et al., 2011); WS (Schülke, Smetana, 2015).

*Brachygluta haematica* (Reichenbach, 1816)

Tyumen Prov. (Bukhkalo et al., 2011); WS (Schülke, Smetana, 2015).

**Genus** *Rybaxis* Saulcy, 1876

*Rybaxis longicornis* (Leach, 1817)

Tyumen Prov. (Bukhkalo et al., 2011); WS (Schülke, Smetana, 2015).

**Tribe** *Euplectini* Streubel, 1839

**Genus** *Euplectus* Leach, 1817

*Euplectus brunneus* (Grimmer, 1841)

Sverdlovsk Prov. (Krasutsky, 1995, 2005); Tyumen Prov. (Bukhkalo et al., 2011; Krasutsky, 1997, 2005).

*Euplectus karstenii* (Reichenbach, 1816)

WS (Schülke, Smetana, 2015).

*Euplectus kirbii* Denny, 1825

WS (Schülke, Smetana, 2015).

**Tribe** *Trichonychini* Reitter, 1882

**Genus** *Bibloplectus* Reitter, 1882

*Bibloplectus ambiguous,* Reichenbach, 1816

Tyumen Prov. (Bukhkalo et al., 2011).

**Genus** *Trimium* Aube, 1833

*Trimium brevicorne* (Reichenbach, 1816)

Tyumen Prov. (Bukhkalo et al., 2011).

**Tribe** *Bythinini* Raffray, 1890

**Genus** *Bryaxis* Kugelann, 1794

*Bryaxis bulbifer* (Reichenbach, 1816)

Tyumen Prov. (Bukhkalo et al., 2011); WS (Schülke, Smetana, 2015).

**Genus** *Bythinus* Leach, 1817

*Bythinus macropalpus* Aube, 1833

Tyumen Prov. (Bukhkalo et al., 2011).

**Tribe** *Pselaphini* Latreille, 1802

**Genus** *Pselaphaulax* Reitter, 1909

*Pselaphaulax dresdensis* (Herbst, 1791)

WS (Schülke, Smetana, 2015).

**Genus** *Pselaphus* Herbst, 1791

*Pselaphus heisei* Herbst, 1791

Tyumen Prov. (Bukhkalo et al., 2011); WS (Schülke, Smetana, 2015).

**Tribe** *Tyrini* Reitter, 1882

**Genus** *Tyrus* Aube, 1833

*Tyrus mucronatus* (Panzer, 1803)

WS (Schülke, Smetana, 2015).

**Subfamily *Scaphidiinae* Latreille, 1806**

**Tribe** *Scaphidiini* Latreille, 1806

**Genus** *Scaphidium* Olivier, 1790

*Scaphidium quadrimaculatum* Olivier, 1790

Kurgan Prov. (Krasutsky, 2005); Sverdlovsk Prov (Krasutsky, 2005); Tyumen Prov. (Bukhkalo et al., 2011); WS (Schülke, Smetana, 2015).

**Subfamily *Scaphidiinae* Latreille, 1806**

**Tribe** *Scaphisomatini* Casey, 1894

**Genus** *Scaphisoma* Leach, 1815

*Scaphisoma agaricinum* (Linnaeus, 1758)

Kurgan Prov. (Krasutsky, 2005); Sverdlovsk Prov (Krasutsky, 1995, 2005); Tyumen Prov. (Bukhkalo et al., 2011; Krasutsky, 2005); WS (Schülke, Smetana, 2015).

*Scaphisoma assimile* Erichson, 1845

Kurgan Prov. (Krasutsky, 2005); Sverdlovsk Prov (Krasutsky, 2005).

*Scaphisoma boleti* (Panzer, 1793)

WS (Schülke, Smetana, 2015).

*Scaphisoma boreale* Lundblad, 1952

WS (Schülke, Smetana, 2015).

*Scaphisoma inopinatum* Löbl, 1967

Khanty-Mansi Distr. (Krasutsky, 1997, 2005); Kurgan Prov. (Krasutsky, 2005); Sverdlovsk Prov (Krasutsky, 2005); Tyumen Prov. (Bukhkalo et al., 2011; Krasutsky, 2005); WS (Schülke, Smetana, 2015).

*Scaphisoma limbatum* Erichson, 1845

Sverdlovsk Prov. (Krasutsky, 1995); Kurgan Prov. (Krasutsky, 2005); Sverdlovsk Prov (Krasutsky, 2005); Tyumen Prov. (Bukhkalo et al., 2011; Krasutsky, 2005).

*Scaphisoma subalpinum* Reitter, 1880

Khanty-Mansi Distr. (Krasutsky, 1009, 2005); Kurgan Prov. (Krasutsky, 2005); Sverdlovsk Prov (Krasutsky, 2005); Tyumen Prov. (Bukhkalo et al., 2011; Krasutsky, 2005).

**Subfamily *Scydmaeninae* Leach, 1815**

**Tribe** *Eutheiini* Casey, 1897

**Genus** *Eutheia* Stephens, 1830

*Eutheia linearis* Mulsant & Rey, 1861

WS (Schülke, Smetana, 2015).

**Tribe** *Cyrtoscydmini* L.W. Schaufuss, 1889

**Genus** *Euconnus* Thomson, 1859

*Euconnus claviger* (P.W.J. Müller & Kunze, 1822)

WS (Schülke, Smetana, 2015).

**Genus** *Stenichnus* Thomson, 1859

*Stenichnus bicolor* (Denny, 1825)

WS (Schülke, Smetana, 2015).

**Subfamily *Staphylininae* Latreille, 1802**

**Tribe** *Staphylinini* Latreille, 1802

**Genus** *Heterothops* Stephens, 1829

*Heterothops dissimilis* (Gravenhorst, 1802)

Kemerovo Prov. (Babenko, 1982); Novosibirsk Prov. (Pavlov, 2005); Tomsk Prov. (Babenko, 1982); WS (Schülke, Smetana, 2015).

*Heterothops praevius* Erichson, 1839

Novosibirsk Prov. (Pavlov, 2005); WS (Schülke, Smetana, 2015).

*Heterothops quadripunctulus* (Gravenhorst, 1806)

Novosibirsk Prov. (Pavlov, 2005); Tyumen Prov. (Bukhkalo et al., 2011, 2012); WS (Schülke, Smetana, 2015).

*Heterothops tenuiventris* Kirshenblat, 1938

WS (Schülke, Smetana, 2015).

**Genus** *Bisnius* Stephens, 1829

*Bisnius cephalotes* (Gravenhorst, 1802)

Kemerovo Prov. (Zinchenko, 2003); WS (Schülke, Smetana, 2015).

*Bisnius fimetarius* (Gravenhorst, 1802)

Kemerovo Prov. (Babenko, 1982; Zinchenko, 2003); Novosibirsk Prov. (Pavlov, 2005); Tomsk Prov. (Babenko, 1982); WS (Schülke, Smetana, 2015).

*Bisnius friebi* (Bernhauer, 1927)

Khanty-Mansi Distr. (Filippov, 2011).

*Bisnius longicollis* (Bernhauer, 1908)

WS (Schülke, Smetana, 2015).

*Bisnius nigriventris* (Thomson, 1867)

WS (Schülke, Smetana, 2015).

*Bisnius nitidulus* (Gravenhorst, 1802)

Tyumen Prov. (Bukhkalo et al., 2011); WS (Schülke, Smetana, 2015).

*Bisnius puella* (Nordmann, 1837)

WS (Schülke, Smetana, 2015).

*Bisnius scribae* (Fauvel, 1867)

WS (Schülke, Smetana, 2015).

*Bisnius sordidus* (Gravenhorst, 1802)

Novosibirsk Prov. (Pavlov, 2005); WS (Schülke, Smetana, 2015).

*Bisnius spermophili* (Ganglbauer, 1897)

Kemerovo Prov. (Zinchenko, 2003); Novosibirsk Prov. (Pavlov, 2005); WS (Schülke, Smetana, 2015).

**Genus** *Eccoptolonthus* Bernhauer, 1912

*Eccoptolonthus rutiliventris* (Sharp, 1874)

WS (Schülke, Smetana, 2015).

**Genus** *Erichsonius* Fauvel, 1874

*Erichsonius cinerascens* (Gravenhorst, 1802)

Tyumen Prov. (Bukhkalo et al., 2011, 2012).

**Genus** *Gabrius* Stephens, 1829

*Gabrius appendiculatus* Sharp, 1910

Novosibirsk Prov. (Pavlov, 2005); Tyumen Prov. (Bukhkalo et al., 2011, 2012); WS (Schülke, Smetana, 2015).

*Gabrius astutus* (Erichson, 1840)

Novosibirsk Prov. (Pavlov, 2005); WS (Schülke, Smetana, 2015).

*Gabrius austriacus* Scheerpeltz, 1947

Tyumen Prov. (Bukhkalo et al., 2011, 2012); WS (Schülke, Smetana, 2015).

*Gabrius nigritulus* (Gravenhorst, 1802)

Kemerovo Prov. (Babenko, 1982); Novosibirsk Prov. (Pavlov, 2005); Tomsk Prov. (Babenko, 1982); WS (Schülke, Smetana, 2015).

*Gabrius osseticus* (Kolenati, 1846)

Novosibirsk Prov. (Pavlov, 2005); Tyumen Prov. (Bukhkalo et al., 2011, 2012); WS (Schülke, Smetana, 2015).

*Gabrius sphagnicola* (Sjöberg, 1950)

Tyumen Prov. (Bukhkalo et al., 2011, 2012); WS (Schülke, Smetana, 2015).

*Gabrius trossulus* (Nordmann, 1837)

Tyumen Prov. (Bukhkalo et al., 2011, 2012).

**Genus** *Gabronthus* Tottenham, 1955

*Gabronthus thermarum* (Aube, 1850)

Novosibirsk Prov. (Pavlov, 2005); WS (Schülke, Smetana, 2015).

**Genus** *Neobisnius* Ganglbauer, 1895

*Neobisnius procerulus* (Gravenhorst, 1806)

Kemerovo Prov. (Babenko, 1982; Zinchenko, 2003); Novosibirsk Prov. (Pavlov, 2005); Tomsk Prov. (Babenko, 1982); WS (Schülke, Smetana, 2015).

*Neobisnius villosulus* (Stephens, 1833)

Novosibirsk Prov. (Pavlov, 2005).

**Genus** *Philonthus* Stephens, 1829

*Philonthus addendus* Sharp, 1867

Kemerovo Prov. (Babenko, 1982; Zinchenko, 2003); Novosibirsk Prov. (Pavlov, 2005); Tomsk Prov. (Babenko, 1982); Tyumen Prov. (Bukhkalo et al., 2011, 2012); WS (Schülke, Smetana, 2015).

*Philonthus albipes* (Gravenhorst, 1802)

Kemerovo Prov. (Babenko, 1982); Tomsk Prov. (Babenko, 1982); WS (Schülke, Smetana, 2015).

*Philonthus atratus* (Gravenhorst, 1802)

Kemerovo Prov. (Babenko, 1982); Tomsk Prov. (Babenko, 1982).

*Philonthus binotatus* (Gravenhorst, 1806)

Kemerovo Prov. (Babenko, 1982); Khanty-Mansi Distr. (Filippov, 2011); Tomsk Prov. (Babenko, 1982); Tyumen Prov. (Bukhkalo et al., 2011, 2012); WS (Schülke, Smetana, 2015).

*Philonthus carbonarius* (Gravenhorst, 1802)

Kemerovo Prov. (Babenko, 1982; Zinchenko, 2003); Novosibirsk Prov. (Pavlov, 2002); Tomsk Prov. (Babenko, 1982; Babenko, Nuzhnykh, 2014); Tyumen Prov. (Bukhkalo et al., 2011, 2012); WS (Schülke, Smetana, 2015).

*Philonthus caucasicus* Nordmann, 1837

WS (Schülke, Smetana, 2015).

*Philonthus chalceus* Stephens, 1832

Kemerovo Prov. (Babenko, 1982); Tomsk Prov. (Babenko, 1982).

*Philonthus cognatus* Stephens, 1832

Tomsk Prov. (Babenko, Nuzhnykh, 2014); Tyumen Prov. (Bukhkalo et al., 2011, 2012); WS (Schülke, Smetana, 2015).

*Philonthus concinnus* (Gravenhorst, 1802)

Kemerovo Prov. (Babenko, 1982); Novosibirsk Prov. (Pavlov, 2005, 2006); Tomsk Prov. (Babenko, 1982; Babenko, Nuzhnykh, 2014); Tyumen Prov. (Bukhkalo et al., 2011, 2012); WS (Schülke, Smetana, 2015).

**Genus** *Creophilus* Leach, 1819

*Creophilus maxillosus* (Linnaeus, 1758)

Kemerovo Prov. (Babenko, 1982); Novosibirsk Prov. (Pavlov, 2005); Tomsk Prov. (Babenko, 1982); Tyumen Prov. (Bukhkalo et al., 2011, 2012); WS (Schülke, Smetana, 2015).

**Genus** *Dinothenarus* Thomson, 1858

*Dinothenarus fossor* (Scopoli, 1771)

Novosibirsk Prov. (Pavlov, 2005).

*Dinothenarus pubescens* (De Geer, 1774)

Kemerovo Prov. (Babenko, 1982); Tomsk Prov. (Babenko, 1982).

*Dinothenarus sibiricus* (Gebler, 1830)

Kemerovo Prov. (Babenko, 1982); Tomsk Prov. (Babenko, 1982); WS (Schülke, Smetana, 2015).

**Genus** *Ocypus* Leach, 1819

*Ocypus aeneocephalus* (De Geer, 1774)

WS (Schülke, Smetana, 2015).

*Ocypus fulvipennis* Erichson, 1840

Kemerovo Prov. (Babenko, 1982); Novosibirsk Prov. (Pavlov, 2005); Tomsk Prov. (Babenko, 1982); WS (Schülke, Smetana, 2015).

*Ocypus fuscatus* (Gravenhorst, 1802)

Kemerovo Prov. (Babenko, 1982); Tomsk Prov. (Babenko, 1982; Babenko, Nuzhnykh, 2014); Tyumen Prov. (Bukhkalo et al., 2011, 2012); WS (Schülke, Smetana, 2015).

*Ocypus fuscoaeneus* Solsky, 1871

WS (Schülke, Smetana, 2015).

*Ocypus nitens* (Schrank, 1781)

Kemerovo Prov. (Babenko, 1982); Tomsk Prov. (Babenko, 1982).

*Ocypus ophthalmicus* Scopoli, 1763

WS (Schülke, Smetana, 2015).

*Ocypus picipennis* (Fabricius, 1792)

Kemerovo Prov. (Babenko, 1982); Tomsk Prov. (Babenko, 1982); WS (Schülke, Smetana, 2015).

**Genus** *Ontholestes* Ganglbauer, 1895

*Ontholestes murinus* (Linnaeus, 1758)

Kemerovo Prov. (Babenko, 1982); Novosibirsk Prov. (Pavlov, 2005); Tomsk Prov. (Babenko, 1982); Tyumen Prov. (Bukhkalo et al., 2011, 2012); WS (Schülke, Smetana, 2015).

*Ontholestes tessellatus* (Geoffroy, 1785)

Kemerovo Prov. (Babenko, 1982); Khanty-Mansi Distr. (Filippov, 2011); Novosibirsk Prov. (Pavlov, 2005); Tomsk Prov. (Babenko, 1982); Tyumen Prov. (Bukhkalo et al., 2011, 2012).

**Subfamily *Staphylininae* Latreille, 1802**

**Tribe** *Staphylinini* Latreille, 1802

**Genus** *Philonthus* Stephens, 1829

*Philonthus concinnus* (Gravenhorst, 1802)

Novosibirsk Prov. (Pavlov, 2006); Tomsk Prov. (Babenko, Nuzhnykh, 2014); Tyumen Prov. (Bukhkalo et al., 2011, 2012); WS (Schülke, Smetana, 2015).

*Philonthus confinis* A.Strand, 1941

WS (Schülke, Smetana, 2015).

*Philonthus coprophilus* Jarrige, 1949

WS (Schülke, Smetana, 2015).

*Philonthus corruscus* (Gravenhorst, 1802)

Kemerovo Prov. (Babenko, 1982); Tomsk Prov. (Babenko, 1982); WS (Schülke, Smetana, 2015).

*Philonthus corvinus* Erichson, 1839

Novosibirsk Prov. (Pavlov, 2005); Tyumen Prov. (Bukhkalo et al., 2011, 2012); WS (Schülke, Smetana, 2015).

*Philonthus cruentatus* (Gmelin, 1790)

Kemerovo Prov. (Babenko, 1982); Tomsk Prov. (Babenko, 1982); WS (Schülke, Smetana, 2015).

*Philonthus cyanipennis* (Fabricius, 1792)

Kemerovo Prov. (Babenko, 1982); Tomsk Prov. (Babenko, 1982); Tyumen Prov. (Bukhkalo et al., 2011, 2012); WS (Schülke, Smetana, 2015).

*Philonthus debilis* (Gravenhorst, 1802)

Kemerovo Prov. (Babenko, 1982); Tomsk Prov. (Babenko, 1982); Novosibirsk Prov. (Pavlov, 2005, 2006); WS (Schülke, Smetana, 2015).

*Philonthus decorus* (Gravenhorst, 1802)

Kemerovo Prov. (Babenko, 1982, Babenko et at al., 2018); Novosibirsk Prov. (Pavlov, 2002, 2005, 2006); Tomsk Prov. (Babenko, 1982, Babenko, Nuzhnykh, 2014, Babenko et at al., 2018, Kercheva, 2022); Tyumen Prov. (Bukhkalo et al., 2011, 2012); WS (Schülke, Smetana, 2015).

*Philonthus dimidiatipennis* Erichson, 1840

Novosibirsk Prov. (Pavlov, 2005, 2006); WS (Schülke, Smetana, 2015).

*Philonthus discoideus* (Gravenhorst, 1802)

WS (Schülke, Smetana, 2015).

*Philonthus ebeninus* (Gravenhorst, 1802)

Kemerovo Prov. (Babenko, 1982); Tomsk Prov. (Babenko, 1982, Babenko, Nuzhnykh, 2014); WS (Schülke, Smetana, 2015).

*Philonthus ephippium* Nordmann, 1837

Novosibirsk Prov. (Pavlov, 2005); WS (Schülke, Smetana, 2015).

*Philonthus fumarius* (Gravenhorst, 1806)

Tyumen Prov. (Bukhkalo et al., 2011, 2012).

*Philonthus furcifer* Renkonen, 1937

Tyumen Prov. (Bukhkalo et al., 2011, 2012); WS (Schülke, Smetana, 2015).

*Philonthus kiautschauensis* Bernhauer, 1916

WS (Schülke, Smetana, 2015).

*Philonthus laminatus* (Creutzer, 1799)

Tyumen Prov. (Bukhkalo et al., 2011, 2012); WS (Schülke, Smetana, 2015).

*Philonthus latiusculus* Hochhuth, 1851

Kemerovo Prov. (Babenko, 1982); Tomsk Prov. (Babenko, 1982); WS (Schülke, Smetana, 2015).

*Philonthus lederi* Eppelsheim, 1893

Khanty-Mansi Distr. (Filippov, 2011); WS (Schülke, Smetana, 2015).

*Philonthus lepidus* (Gravenhorst, 1802)

Kemerovo Prov. (Babenko, 1982); Novosibirsk Prov. (Pavlov, 2002, 2005, 2006); Tomsk Prov. (Babenko, 1982); Tyumen Prov. (Bukhkalo et al., 2011, 2012); WS (Schülke, Smetana, 2015).

*Philonthus longicornis* Stephens, 1832

Kemerovo Prov. (Babenko, 1982); Novosibirsk Prov. (Pavlov, 2005, 2006); Tomsk Prov. (Babenko, 1982); WS (Schülke, Smetana, 2015).

*Philonthus mannerheimi* Fauvel, 1869

Kemerovo Prov. (Zinchenko, 2003); Tyumen Prov. (Bukhkalo et al., 2011, 2012); WS (Schülke, Smetana, 2015).

*Philonthus marginatus* (O.F.Müller, 1764)

Kemerovo Prov. (Babenko, 1982); Tomsk Prov. (Babenko, 1982); Tyumen Prov. (Bukhkalo et al., 2011, 2012); WS (Schülke, Smetana, 2015).

*Philonthus micans* (Gravenhorst, 1802)

Novosibirsk Prov. (Pavlov, 2006); Tyumen Prov. (Bukhkalo et al., 2011, 2012); WS (Schülke, Smetana, 2015).

*Philonthus nigrita* (Gravenhorst, 1806)

Novosibirsk Prov. (Pavlov, 2002, 2005); Tyumen Prov. (Bukhkalo et al., 2011, 2012); WS (Schülke, Smetana, 2015).

*Philonthus nitidus* (Fabricius, 1787)

Kemerovo Prov. (Babenko, 1982); Novosibirsk Prov. (Pavlov, 2002, 2005); Tomsk Prov. (Babenko, 1982); Tyumen Prov. (Bukhkalo et al., 2011, 2012); WS (Schülke, Smetana, 2015).

*Philonthus parvicornis* (Gravenhorst, 1802)

Kemerovo Prov. (Babenko, 1982); Novosibirsk Prov. (Pavlov, 2002, 2005); Tomsk Prov. (Babenko, 1982); WS (Schülke, Smetana, 2015).

*Philonthus politus* (Linnaeus, 1758)

Kemerovo Prov. (Babenko, 1982); Novosibirsk Prov. (Pavlov, 2002, 2005, 2006); Tomsk Prov. (Babenko, 1982, Babenko, Nuzhnykh, 2014); WS (Schülke, Smetana, 2015).

*Philonthus poppiusi* Bernhauer & K.Schubert, 1914

WS (Schülke, Smetana, 2015).

*Philonthus punctus* (Gravenhorst, 1802)

Kemerovo Prov. (Babenko, 1982); Novosibirsk Prov. (Pavlov, 2005); Tomsk Prov. (Babenko, 1982); WS (Schülke, Smetana, 2015).

*Philonthus quisquiliarius* (Gyllenhal, 1810)

Kemerovo Prov. (Babenko, 1982); Novosibirsk Prov. (Pavlov, 2005, 2006); Tomsk Prov. (Babenko, 1982); Tyumen Prov. (Bukhkalo et al., 2011, 2012); WS (Schülke, Smetana, 2015).

*Philonthus rectangulus* Sharp, 1874

Kemerovo Prov. (Babenko, 1982); Tomsk Prov. (Babenko, 1982).

*Philonthus rotundicollis* (Menetries, 1832)

Kemerovo Prov. (Babenko, 1982); Novosibirsk Prov. (Pavlov, 2005); Tomsk Prov. (Babenko, 1982, Babenko, Nuzhnykh, 2014); Tyumen Prov. (Bukhkalo et al., 2011, 2012); WS (Schülke, Smetana, 2015).

*Philonthus rubripennis* Stephens, 1832

Novosibirsk Prov. (Pavlov, 2005, 2006); WS (Schülke, Smetana, 2015).

*Philonthus rufipes* (Stephens, 1832)

Kemerovo Prov. (Babenko, 1982); Novosibirsk Prov. (Pavlov, 2005); Tomsk Prov. (Babenko, 1982); WS (Schülke, Smetana, 2015).

*Philonthus salinus* Kiesenwetter, 1844

Novosibirsk Prov. (Pavlov, 2005); WS (Schülke, Smetana, 2015).

*Philonthus sanguinolentus* (Gravenhorst, 1802)

Kemerovo Prov. (Babenko, 1982); Novosibirsk Prov. (Pavlov, 2002, 2005); Tomsk Prov. (Babenko, 1982); Tyumen Prov. (Bukhkalo et al., 2014); WS (Schülke, Smetana, 2015).

*Philonthus splendens* (Fabricius, 1792)

Kemerovo Prov. (Babenko, 1982); Tomsk Prov. (Babenko, 1982); WS (Schülke, Smetana, 2015).

*Philonthus subvirescens* Thomson, 1884

Tyumen Prov. (Bukhkalo et al., 2011, 2012); WS (Schülke, Smetana, 2015).

*Philonthus succicola* Thomson, 1860

Tyumen Prov. (Bukhkalo et al., 2011, 2012); WS (Schülke, Smetana, 2015).

*Philonthus tenuicornis* Mulsant & Rey, 1853

Novosibirsk Prov. (Pavlov, 2005, 2006); Tyumen Prov. (Bukhkalo et al., 2011, 2012); WS (Schülke, Smetana, 2015).

*Philonthus transbaicalia* Hochhuth, 1851

Kemerovo Prov. (Babenko, 1982); Tomsk Prov. (Babenko, 1982); WS (Schülke, Smetana, 2015).

*Philonthus umbratilis* (Gravenhorst, 1802)

Kemerovo Prov. (Babenko, 1982); Novosibirsk Prov. (Pavlov, 2005, 2006); Tomsk Prov. (Babenko, 1982); Tyumen Prov. (Bukhkalo et al., 2011, 2012).

*Philonthus varians* (Paykull, 1789)

Kemerovo Prov. (Babenko, 1982); Tomsk Prov. (Babenko, 1982); Tyumen Prov. (Bukhkalo et al., 2011, 2012); WS (Schülke, Smetana, 2015).

*Philonthus ventralis* (Gravenhorst, 1802)

Novosibirsk Prov. (Pavlov, 2005, 2006); WS (Schülke, Smetana, 2015).

*Philonthus virgo* (Gravenhorst, 1802)

Novosibirsk Prov. (Pavlov, 2005).

**Genus** *Platydracus* Thomson, 1858

*Platydracus fulvipes* Scopoli, 1763

Khanty-Mansi Distr. (Filippov, 2011); Tyumen Prov. (Bukhkalo et al., 2011, 2012); WS (Schülke, Smetana, 2015).

*Platydracus latebricola* (Gravenhorst, 1806)

Tyumen Prov. (Bukhkalo et al., 2011, 2012); WS (Schülke, Smetana, 2015).

*Platydracus stercorarius* (A. G. Olivier, 1795)

Kemerovo Prov. (Babenko, 1982); Novosibirsk Prov. (Pavlov, 2005); Tomsk Prov. (Babenko, 1982); Tyumen Prov. (Bukhkalo et al., 2011, 2012); WS (Schülke, Smetana, 2015).

**Genus** *Quedionuchus* Sharp, 1884

*Quedionuchus plagiatus* (Mannerheim, 1843)

Khanty-Mansi Distr. (Filippov, 2011); WS (Schülke, Smetana, 2015).

*Quedionuchus glaber* (O.F. Müller, 1776)

Kemerovo Prov. (Babenko, 1982); Tomsk Prov. (Babenko, 1982).

**Genus** *Quedius* Stephens, 1829

*Quedius altaicus* Korge, 1962

WS (Schülke, Smetana, 2015).

*Quedius boopoides* Münster, 1923

Tyumen Prov. (Bukhkalo et al., 2011, 2012); WS (Schülke, Smetana, 2015).

*Quedius boops* (Gravenhorst, 1802)

Tomsk Prov. (Babenko, Nuzhnykh, 2014); Tyumen Prov. (Bukhkalo et al., 2011); WS (Schülke, Smetana, 2015).

*Quedius brevis* Erichson, 1840

Kemerovo Prov. (Babenko, 1982); Tomsk Prov. (Babenko, 1982).

*Quedius centrasiaticus* Coiffait, 1969

WS (Schülke, Smetana, 2015).

*Quedius dilatatus* (Fabricius, 1787)

Tyumen Prov. (Bukhkalo et al., 2011, 2012); WS (Schülke, Smetana, 2015).

*Quedius fellmani* (Zetterstedt, 1838)

WS (Schülke, Smetana, 2015); Yamalo-Nenets Distr. (Olshwang, 1992).

*Quedius fulgidus* (Fabricius, 1792)

Kemerovo Prov. (Babenko, 1982); Tomsk Prov. (Babenko, 1982).

*Quedius fuliginosus* (Gravenhorst, 1802)

Kemerovo Prov. (Babenko, 1982); Tomsk Prov. (Babenko, 1982, Babenko, Nuzhnykh, 2014); Tyumen Prov. (Bukhkalo et al., 2011, 2012); WS (Schülke, Smetana, 2015).

*Quedius fulvicollis* (Stephens, 1833)

Tyumen Prov. (Bukhkalo et al., 2011, 2012); WS (Schülke, Smetana, 2015).

*Quedius jenisseensis* (J. Sahlberg, 1880)

Tyumen Prov. (Bukhkalo et al., 2011, 2012); Yamalo-Nenets Distr. (Smetana, Shavrin. 2018); WS (Schülke, Smetana, 2015),

*Quedius limbatus* (Heer, 1839)

Tyumen Prov. (Bukhkalo et al., 2011, 2012).

*Quedius longicornis* Kraatz, 1857

Tyumen Prov. (Bukhkalo et al., 2011, 2012); WS (Schülke, Smetana, 2015).

*Quedius mesomelinus* (Marsham, 1802)

WS (Schülke, Smetana, 2015).

*Quedius microps* Gravenhorst, 1847

Kemerovo Prov. (Zinchenko, 2003); Novosibirsk Prov. (Pavlov, 2005); WS (Schülke, Smetana, 2015).

*Quedius molochinus* (Gravenhorst, 1806)

Khanty-Mansi Distr. (Filippov, 2011); Novosibirsk Prov. (Pavlov, 2005); Tyumen Prov. (Bukhkalo et al., 2011, 2012); WS (Schülke, Smetana, 2015).

*Quedius ochripennis* (Menetries, 1832)

Novosibirsk Prov. (Pavlov, 2005); WS (Schülke, Smetana, 2015).

*Quedius paraboops* Coiffait, 1975

WS (Schülke, Smetana, 2015).

*Quedius picipes* (Mannerheim, 1830)

Novosibirsk Prov. (Pavlov, 2005); WS (Schülke, Smetana, 2015).

*Quedius puncticollis* (C. G. Thomson, 1867)

Kemerovo Prov. (Zinchenko, 2003); Tyumen Prov. (Bukhkalo et al., 2011, 2012); WS (Schülke, Smetana, 2015).

*Quedius sublimbatus* Mäklin, 1853

WS (Schülke, Smetana, 2015).

*Quedius tenellus* (Gravenhorst, 1806)

Kemerovo Prov. (Babenko, 1982); Tomsk Prov. (Babenko, 1982); WS (Schülke, Smetana, 2015).

**Genus** *Rabigus* Mulsant & Rey, 1876

*Rabigus pullus* (Nordmann, 1837)

WS (Schülke, Smetana, 2015).

*Rabigus tenuis* (Fabricius, 1792)

Novosibirsk Prov. (Pavlov, 2005); WS (Schülke, Smetana, 2015).

**Genus** *Staphylinus* Linnaeus, 1758

*Philonthus rubripennis* Stephens, 1832

Kemerovo Prov. (Babenko, 1982); Tomsk Prov. (Babenko, 1982).

*Platydracus stercorarius* (A. G. Olivier, 1795)

Tomsk Prov. (Babenko, Nuzhnykh, 2014).

*Staphylinus erythropterus* Linnaeus, 1758

Kemerovo Prov. (Babenko, 1982); Novosibirsk Prov. (Pavlov, 2005); Tomsk Prov. (Babenko, 1982); Tyumen Prov. (Bukhkalo et al., 2011, 2012, Vazhenina, 2019); WS (Schülke, Smetana, 2015).

**Genus** *Tasgius* Stephens, 1829

*Tasgius morsitans* (P. Rossi, 1790)

Novosibirsk Prov. (Pavlov, 2005); WS (Schülke, Smetana, 2015).

**Subfamily *Steninae* Macleay, 1825**

**Genus** *Dianous* Leach, 1819

*Dianous coerulescens* (Gyllenhal, 1810)

WS (Schülke, Smetana, 2015).

**Genus** *Stenus* Latreille, 1797

*Stenus aereus* Solsky, 1871

WS (Schülke, Smetana, 2015).

*Stenus altaicus* Puthz, 1984

WS (Schülke, Smetana, 2015).

*Stenus argus* Gravenhorst, 1806

Tyumen Prov. (Bukhkalo et al., 2011, 2012); WS (Schülke, Smetana, 2015).

*Stenus assequens* Rey, 1884

Novosibirsk Prov. (Pavlov, 2005).

*Stenus aterrimus* Erichson, 1839

WS (Schülke, Smetana, 2015).

*Stenus auriger* Eppelsheim, 1893

Khanty-Mansi Distr. (Filippov, 2011).

*Stenus baicalensis* Puthz, 1968

Novosibirsk Prov. (Pavlov, 2005); WS (Schülke, Smetana, 2015).

*Stenus bifoveolatus* Gyllenhal, 1827

Tyumen Prov. (Bukhkalo et al., 2011).

*Stenus biguttatus* (Linnaeus, 1758)

Kemerovo Prov. (Babenko, 1982); Tomsk Prov. (Babenko, 1982); Tyumen Prov. (Bukhkalo et al., 2011, 2012); WS (Schülke, Smetana, 2015).

*Stenus bilineatus* J. Sahlberg, 1871

Tyumen Prov. (Bukhkalo et al., 2011).

*Stenus bimaculatus* Gyllenhal, 1810

Kemerovo Prov. (Babenko, 1982); Tomsk Prov. (Babenko, 1982); Tyumen Prov. (Bukhkalo et al., 2011, 2012); WS (Schülke, Smetana, 2015).

*Stenus binotatus* Ljungh, 1804

WS (Schülke, Smetana, 2015).

*Stenus bohemicus* Machulka, 1947

Khanty-Mansi Distr. (Filippov, 2011); Novosibirsk Prov. (Pavlov, 2005); Tyumen Prov. (Bukhkalo et al., 2011); WS (Schülke, Smetana, 2015).

*Stenus boops* Ljungh, 1810

Kemerovo Prov. (Babenko, 1982); Khanty-Mansi Distr. (Filippov, 2011); Tomsk Prov. (Babenko, 1982); Tyumen Prov. (Bukhkalo et al., 2011, 2012); WS (Schülke, Smetana, 2015).

*Stenus calcaratus* W.Scriba, 1865

Khanty-Mansi Distr. (Filippov, 2011); Tyumen Prov. (Bukhkalo et al., 2011, 2012); WS (Schülke, Smetana, 2015).

*Stenus canaliculatus* Gyllenhal, 1827

Altai Terr. (Ryvkin, 2012); Novosibirsk Prov. (Pavlov, 2005); Tyumen Prov. (Bukhkalo et al., 2012); WS (Schülke, Smetana, 2015).

*Stenus carbonarius* Gyllenhal, 1827

Novosibirsk Prov. (Pavlov, 2005); Tyumen Prov. (Bukhkalo et al., 2011); WS (Schülke, Smetana, 2015).

*Stenus cautus* Erichson, 1839

Omsk Prov. (Ryvkin, 2014); WS (Schülke, Smetana, 2015).

*Stenus cephallenicus* Bernhauer, 1913

WS (Schülke, Smetana, 2015).

*Stenus cicindeloides* (Schaller, 1783)

Novosibirsk Prov. (Pavlov, 2005); Tyumen Prov. (Bukhkalo et al., 2011); WS (Schülke, Smetana, 2015).

*Stenus circularis* Gravenhorst, 1802

Tyumen Prov. (Bukhkalo et al., 2011, 2012); WS (Schülke, Smetana, 2015).

*Stenus clavicornis* (Scopoli, 1763)

Kemerovo Prov. (Babenko, 1982); Novosibirsk Prov. (Pavlov, 2005); Tomsk Prov. (Babenko, 1982); Tyumen Prov. (Bukhkalo et al., 2011, 2012); WS (Schülke, Smetana, 2015).

*Stenus comma* LeConte, 1863

Kemerovo Prov. (Babenko, 1982); Khanty-Mansi Distr. (Filippov, 2011); Novosibirsk Prov. (Pavlov, 2005); Tomsk Prov. (Babenko, 1982); Tyumen Prov. (Bukhkalo et al., 2011, 2012); WS (Schülke, Smetana, 2015).

*Stenus confusus* J.Sahlberg, 1876

Yamalo-Nenets Distr. (Ryvkin, 2012); WS (Schülke, Smetana, 2015).

*Stenus crassus* Stephens, 1833

Novosibirsk Prov. (Pavlov, 2005); WS (Schülke, Smetana, 2015).

*Stenus europaeus* Puthz, 1966

Khanty-Mansi Distr. (Ryvkin, 2014, Filippov, 2011); Tyumen Prov. (Bukhkalo et al., 2011, 2012, Ryvkin, 2014); WS (Schülke, Smetana, 2015).

*Stenus excubitor* Erichson, 1839

Tyumen Prov. (Bukhkalo et al., 2011); WS (Schülke, Smetana, 2015).

*Stenus exspectatus*Puthz, 1965

Tyumen Prov. (Bukhkalo et al., 2011, 2012); WS (Schülke, Smetana, 2015).

*Stenus flavipalpis*Thomson, 1860

Tyumen Prov. (Bukhkalo et al., 2011)

*Stenus fornicatus*Stephens, 1833

WS (Schülke, Smetana, 2015).

*Stenus fossulatus*Erichson, 1840

Tyumen Prov. (Bukhkalo et al., 2011)

*Stenus fuscipes*Gravenhorst, 1802

Tyumen Prov. (Bukhkalo et al., 2011, 2012); WS (Schülke, Smetana, 2015).

*Stenus gibbicollis*J.Sahlberg, 1880

WS (Schülke, Smetana, 2015).

*Stenus glabellus* Thomson, 1870

Tyumen Prov. (Bukhkalo et al., 2011).

*Stenus humboldti*Puthz, 1984

WS (Schülke, Smetana, 2015).

*Stenus humilis*Erichson, 1839

Novosibirsk Prov. (Pavlov, 2005); Tyumen Prov. (Bukhkalo et al., 2011, 2012); WS (Schülke, Smetana, 2015).

*Stenus hyperboreus*J.Sahlberg, 1876

WS (Schülke, Smetana, 2015).

*Stenus hypoproditor*Puthz, 1965

Novosibirsk Prov. (Pavlov, 2005); WS (Schülke, Smetana, 2015).

*Stenus immarginatus*Mäklin, 1853

WS (Schülke, Smetana, 2015).

*Stenus incrassatus*Erichson, 1839

Novosibirsk Prov. (Pavlov, 2005); Tyumen Prov. (Bukhkalo et al., 2011); WS (Schülke, Smetana, 2015).

*Stenus jacuticus*Poppius, 1909

WS (Schülke, Smetana, 2015).

*Stenus juno*(Paykull, 1789)

Khanty-Mansi Distr. (Filippov, 2011); Novosibirsk Prov. (Pavlov, 2005); Tyumen Prov. (Bukhkalo et al., 2011, 2012); WS (Schülke, Smetana, 2015).

*Stenus kiesenwetteri*Rosenhauer, 1856

Tyumen Prov. (Bukhkalo et al., 2011).

*Stenus kolbei*Gerhardt, 1893

Tyumen Prov. (Bukhkalo et al., 2011, 2012); WS (Schülke, Smetana, 2015).

*Stenus kongsbergensis*Münster, 1911

WS (Schülke, Smetana, 2015).

**Genus** *Stenus labilis*Erichson, 1840

Khanty-Mansi Distr. (Filippov, 2011); Tyumen Prov. (Bukhkalo et al., 2011); WS (Schülke, Smetana, 2015).

*Stenus lagopodis*Ryvkin, 1987

Khanty-Mansi Distr. (Filippov, 2011).

*Stenus latifrons*Erichson, 1839

Tyumen Prov. (Bukhkalo et al., 2011).

*Stenus latipennis*J. Sahlberg, 1880

WS (Schülke, Smetana, 2015); Yamalo-Nenets Distr. (Ryvkin, 2012).

*Stenus ludyi*Fauvel, 1886

Tyumen Prov. (Bukhkalo et al., 2011, 2012); WS (Schülke, Smetana, 2015).

*Stenus lustrator*Erichson, 1839

Tyumen Prov. (Bukhkalo et al., 2011, 2012); WS (Schülke, Smetana, 2015).

*Stenus melanarius*Stephens, 1833

Tyumen Prov. (Bukhkalo et al., 2011).

*Stenus morio*Gravenhorst, 1806

Tyumen Prov. (Bukhkalo et al., 2011); WS (Schülke, Smetana, 2015).

*Stenus nanus*Stephens, 1833

Novosibirsk Prov. (Pavlov, 2005); Tyumen Prov. (Bukhkalo et al., 2011, 2012); WS (Schülke, Smetana, 2015).

*Stenus nigritulus*Gyllenhal, 1827

Novosibirsk Prov. (Pavlov, 2005); Tyumen Prov. (Bukhkalo et al., 2011, 2012); WS (Schülke, Smetana, 2015).

*Stenus nitens*Stephens, 1833

Khanty-Mansi Distr. (Ryvkin, 2012); WS (Schülke, Smetana, 2015).

*Stenus noctivagus*Casey, 1884

WS (Schülke, Smetana, 2015).

*Stenus opticus*Gravenhorst, 1806

Tyumen Prov. (Bukhkalo et al., 2011, 2012; Vazhenina, 2019); WS (Schülke, Smetana, 2015).

*Stenus pallipes*Gravenhorst, 1802

Tyumen Prov. (Bukhkalo et al., 2011, 2012); WS (Schülke, Smetana, 2015).

*Stenus pallitarsis*Stephens, 1833

Tyumen Prov. (Bukhkalo et al., 2011, 2012); WS (Schülke, Smetana, 2015).

*Stenus palposus*Zetterstedt, 1838

WS (Schülke, Smetana, 2015).

*Stenus palustris*Erichson, 1839

Tyumen Prov. (Bukhkalo et al., 2011, 2012); WS (Schülke, Smetana, 2015).

*Stenus permundus*Ryvkin, 2002

WS (Schülke, Smetana, 2015).

*Stenus proditor*Erichson, 1839

WS (Schülke, Smetana, 2015).

*Stenus ageus*Casey, 1884

Khanty-Mansi Distr. (Filippov, 2011).

*Stenus rigidus*Casey, 1884

Khanty-Mansi Distr. (Ryvkin, 2014); Tyumen Prov. (Ryvkin, 2014); Yamalo-Nenets Distr. (Ryvkin, 2014).

*Stenus ageus*Casey, 1884

WS (Schülke, Smetana, 2015).

*Stenus rufomaculatus* Bernhauer, 1902

WS (Schülke, Smetana, 2015).

*Stenus ruralis*Erichson, 1840

Novosibirsk Prov. (Pavlov, 2005); Tyumen Prov. (Bukhkalo et al., 2011, 2012); WS (Schülke, Smetana, 2015).

*Stenus semiputatus*Ryvkin, 1995

WS (Schülke, Smetana, 2015).

*Stenus sibiricus*J. Sahlberg, 1880

Khanty-Mansi Distr. (Filippov, 2011); Tyumen Prov. (Ryvkin, 2014); Yamalo-Nenets Distr. (Ольшванг, 1992).

*Stenus similis*(Herbst, 1784)

WS (Schülke, Smetana, 2015).

*Stenus strandi*L. Benick, 1937

Khanty-Mansi Distr. (Filippov, 2011); WS (Schülke, Smetana, 2015).

*Stenus sylvester*Erichson, 1839

Tyumen Prov. (Bukhkalo et al., 2011).

*Stenus tarsalis*Ljungh, 1810

Kemerovo Prov. (Babenko, 1982); Tomsk Prov. (Babenko, 1982); Khanty-Mansi Distr. (Filippov, 2011); WS (Schülke, Smetana, 2015).

*Stenus veselovae*Ryvkin, 1987

WS (Schülke, Smetana, 2015).

*Stenus wuesthoffi*L. Benick, 1941

Khanty-Mansi Distr. (Filippov, 2011); Tyumen Prov. (Bukhkalo et al., 2011); WS (Schülke, Smetana, 2015).

**Subfamily *Tachyporinae* Macleay, 1825**

**Genus** *Cilea* Jacquelin du Val, 1856

*Cilea silphoides*(Linnaeus, 1767)

Kurgan Prov. (Krasutsky, 2005); Sverdlovsk Prov (Krasutsky, 2005); Tyumen Prov.(Krasutsky, 2005); WS (Schülke, Smetana, 2015).

**Genus** *Lamprinodes* Luze, 1901

*Lamprinodes saginatus*(Gravenhorst, 1806)

Tyumen Prov. (Bukhkalo et al., 2011, 2012).

**Genus** *Sepedophilus* Gistel, 1856

*Sepedophilus bipustulatus*(Gravenhorst, 1802)

Khanty-Mansi Distr. (Krasutsky, 1997, 2005); Kurgan Prov. (Krasutsky, 2005); Sverdlovsk Prov. (Krasutsky, 1995, 2005); Tyumen Prov. (Bukhkalo et al., 2011, 2012; Krasutsky, 2005); WS (Schülke, Smetana, 2015).

*Sepedophilus constans*(Fowler, 1888)

Tyumen Prov. (Bukhkalo et al., 2011, 2012); WS (Schülke, Smetana, 2015).

*Sepedophilus immaculatus*(Stephens, 1832)

Tyumen Prov. (Bukhkalo et al., 2011, 2012); WS (Schülke, Smetana, 2015).

*Sepedophilus littoreus*(Linnaeus, 1758)

Kemerovo Prov. (Babenko, 1982); Tomsk Prov. (Babenko, 1982); WS (Schülke, Smetana, 2015).

*Sepedophilus marshami*(Stephens, 1832)

Tyumen Prov. (Bukhkalo et al., 2011, 2012).

*Sepedophilus pedicularius*(Gravenhorst, 1802)

Kemerovo Prov. (Babenko, 1982); Tomsk Prov. (Babenko, 1982); Tyumen Prov. (Bukhkalo et al., 2011, 2012); WS (Schülke, Smetana, 2015).

*Sepedophilus testaceus*(Fabricius, 1792)

Kemerovo Prov. (Babenko, 1982); Khanty-Mansi Distr. (Filippov, 2011); Tomsk Prov. (Babenko, 1982); Tyumen Prov. (Bukhkalo et al., 2011, 2012); WS (Schülke, Smetana, 2015).

**Genus** *Tachinus* Gravenhorst, 1802

*Tachinus absconditus*Luze, 1900

WS (Schülke, Smetana, 2015).

*Tachinus arcticus*(Motschulsky, 1860)

Khanty-Mansi Distr. (Zinovyev et al., 2016).

*Tachinus basalis*Erichson, 1839

WS (Schülke, Smetana, 2015).

*Tachinus bernhaueri*Luze, 1901

WS (Schülke, Smetana, 2015).

*Tachinus bicuspidatus*J.Sahlberg, 1880

Kemerovo Prov. (Babenko, 1982); Tomsk Prov. (Babenko, 1982); Tyumen Prov. (Bukhkalo et al., 2011, 2012); WS (Schülke, Smetana, 2015).

*Tachinus brevipennis*J.Sahlberg, 1880

Khanty-Mansi Distr. (Zinovyev et al., 2016).

*Tachinus collaris*Gravenhorst, 1802

Kemerovo Prov. (Babenko, 1982); Tomsk Prov. (Babenko, 1982).

*Tachinus corticinus*Gravenhorst, 1802

Tyumen Prov. (Bukhkalo et al., 2011, 2012); WS (Schülke, Smetana, 2015).

*Tachinus discoideus*Erichson, 1839

Kemerovo Prov. (Babenko, 1982); Tomsk Prov. (Babenko, 1982).

*Tachinus elongatus*Gyllenhal, 1810

Kemerovo Prov. (Babenko, 1982); Khanty-Mansi Distr. (Filippov, 2011); Tomsk Prov. (Babenko, 1982); Tyumen Prov. (Bukhkalo et al., 2011, 2012); WS (Schülke, Smetana, 2015).

*Tachinus fimetarius*Gravenhorst, 1802

Kemerovo Prov. (Babenko, 1982); Novosibirsk Prov. (Pavlov, 2005); Tomsk Prov. (Babenko, 1982); WS (Schülke, Smetana, 2015).

*Tachinus instabilis*Mäklin, 1853

WS (Schülke, Smetana, 2015).

*Tachinus laticollis*Gravenhorst, 1802

Kemerovo Prov. (Babenko, 1982); Khanty-Mansi Distr. (Filippov, 2011); Novosibirsk Prov. (Pavlov, 2005); Sverdlovsk Prov. (Krasutsky, 1995, 2005); Tomsk Prov. (Babenko, 1982; Babenko, Nuzhnykh, 2014); Tyumen Prov. (Bukhkalo et al., 2011, 2012, Krasutsky, 2005); WS (Schülke, Smetana, 2015).

*Tachinus lignorum*(Linnaeus, 1758)

Kemerovo Prov. (Babenko, 1982); Tomsk Prov. (Babenko, 1982); WS (Schülke, Smetana, 2015).

*Tachinus marginatus*(Fabricius, 1792)

Kemerovo Prov. (Babenko, 1982); Tomsk Prov. (Babenko, 1982, Babenko; Nuzhnykh, 2014); Tyumen Prov. (Bukhkalo et al., 2011, 2012); WS (Schülke, Smetana, 2015).

*Tachinus marginellus*(Fabricius, 1781)

Kemerovo Prov. (Babenko, 1982); Khanty-Mansi Distr. (Filippov, 2011); Novosibirsk Prov. (Pavlov, 2005); Tomsk Prov. (Babenko, 1982); Tyumen Prov. (Bukhkalo et al., 2011, 2012); WS (Schülke, Smetana, 2015).

*Tachinus pallipes*(Gravenhorst, 1806)

Kemerovo Prov. (Babenko, 1982); Khanty-Mansi Distr. (Filippov, 2011); Tomsk Prov. (Babenko, 1982); Tyumen Prov. (Bukhkalo et al., 2011, 2012); WS (Schülke, Smetana, 2015).

*Tachinus proximus*Kraatz, 1855

WS (Schülke, Smetana, 2015).

*Tachinus punctipennis*(J. Sahlberg, 1876)

WS (Schülke, Smetana, 2015).

*Tachinus rufipennis*Gyllenhal, 1810

Novosibirsk Prov. (Pavlov, 2005); WS (Schülke, Smetana, 2015).

*Tachinus rufipes*(Linnaeus, 1758)

Kemerovo Prov. (Babenko, 1982); Novosibirsk Prov. (Pavlov, 2005); Tomsk Prov. (Babenko, 1982; Babenko, Nuzhnykh, 2014); Tyumen Prov. (Bukhkalo et al., 2011, 2012; Vazhenina, 2019).

*Tachinus rufitarsis* Hochhuth, 1849 г.

WS (Schülke, Smetana, 2015).

*Tachinus scapularis*Stephens, 1832

WS (Schülke, Smetana, 2015).

*Tachinus splendens*Bernhauer, 1903

WS (Schülke, Smetana, 2015).

**Genus** *Tachyporus* Gravenhorst, 1802

*Tachyporus abdominalis*(Fabricius, 1782)

Kemerovo Prov. (Babenko, 1982); Novosibirsk Prov. (Pavlov, 2005); Tomsk Prov. (Babenko, 1982; Babenko, Nuzhnykh, 2014); Tyumen Prov. (Bukhkalo et al., 2011, 2012); WS (Schülke, Smetana, 2015).

*Tachyporus atriceps*Stephens, 1832

Tyumen Prov. (Bukhkalo et al., 2011, 2012); WS (Schülke, Smetana, 2015).

*Tachyporus chrysomelinus*(Linnaeus, 1758)

Kemerovo Prov. (Babenko, 1982); Novosibirsk Prov. (Pavlov, 2005); Tomsk Prov. (Babenko, 1982); Tyumen Prov. (Bukhkalo et al., 2011, 2012; Vazhenina, 2019); WS (Schülke, Smetana, 2015).

*Tachyporus corpulentus*J. Sahlberg, 1876

Tyumen Prov. (Bukhkalo et al., 2011, 2012); WS (Schülke, Smetana, 2015).

*Tachyporus dispar*(Paykull, 1789)

Tyumen Prov. (Bukhkalo et al., 2011, 2012); WS (Schülke, Smetana, 2015).

*Tachyporus formosus*A.H. Matthews, 1838

Kemerovo Prov. (Babenko, 1982); Novosibirsk Prov. (Pavlov, 2005); Tomsk Prov. (Babenko, 1982); WS (Schülke, Smetana, 2015).

*Tachyporus hypnorum*(Fabricius, 1775)

Kemerovo Prov. (Babenko, 1982); Tomsk Prov. (Babenko, 1982); WS (Schülke, Smetana, 2015).

*Tachyporus nitidulus*(Fabricius, 1781)

Kemerovo Prov. (Babenko, 1982); Khanty-Mansi Distr. (Filippov, 2011); Novosibirsk Prov. (Pavlov, 2005); Tomsk Prov. (Babenko, 1982); Tyumen Prov. (Bukhkalo et al., 2011, 2012); WS (Schülke, Smetana, 2015).

*Tachyporus obscurellus*Zetterstedt, 1838

Tyumen Prov. (Bukhkalo et al., 2011, 2012); WS (Schülke, Smetana, 2015).

*Tachyporus obtusus*(Linnaeus, 1767)

Kemerovo Prov. (Babenko, 1982); Khanty-Mansi Distr. (Filippov, 2011); Novosibirsk Prov. (Pavlov, 2005); Tomsk Prov. (Babenko, 1982); Tyumen Prov. (Bukhkalo et al., 2011, 2012); WS (Schülke, Smetana, 2015).

*Tachyporus pallidus*Sharp, 1871

Tyumen Prov. (Bukhkalo et al., 2012); WS (Schülke, Smetana, 2015).

*Tachyporus pulchellus*Mannerheim, 1843

Tyumen Prov. (Bukhkalo et al., 2011, 2012; Vazhenina, 2019); WS (Schülke, Smetana, 2015).

*Tachyporus pusillus*Gravenhorst, 1806

Kemerovo Prov. (Babenko, 1982); Novosibirsk Prov. (Pavlov, 2005); Tomsk Prov. (Babenko, 1982; Babenko, Nuzhnykh, 2014); Tyumen Prov. (Bukhkalo et al., 2011, 2012).

*Tachyporus quadriscopulatus*Pandellé, 1869

Tyumen Prov. (Bukhkalo et al., 2011, 2012); WS (Schülke, Smetana, 2015).

*Tachyporus scitulus*Erichson, 1839

Tyumen Prov. (Bukhkalo et al., 2011, 2012).

*Tachyporus solutus*Erichson, 1839

Novosibirsk Prov. (Pavlov, 2005); Tyumen Prov. (Bukhkalo et al., 2011, 2012); WS (Schülke, Smetana, 2015).

*Tachyporus terminalis*Sharp, 1888

WS (Schülke, Smetana, 2015).

*Tachyporus tersus*Erichson, 1839

Tyumen Prov. (Bukhkalo et al., 2011, 2012); WS (Schülke, Smetana, 2015).

*Tachyporus transversalis*Gravenhorst, 1806

Tyumen Prov. (Bukhkalo et al., 2012).

**Subfamily *Trichophyinae*** Thomson, 1858

**Genus** *Trichophya* Mannerheim, 1830

*Trichophya pilicornis*(Gyllenhal, 1810)

WS (Schülke, Smetana, 2015).

**Subfamily *Xantholininae* Erichson, 1839**

**Tribe** *Othiini* Thomson, 1859

**Genus** *Atrecus* Jacquelin du Val, 1856

*Atrecus pilicornis (Paykull, 1790)*

Kemerovo Prov. (Babenko, 1982); Tomsk Prov. (Babenko, 1982); WS (Schülke, Smetana, 2015).

**Genus** *Othius* Stephens, 1829

*Othius angustus*Stephens, 1833

Khanty-Mansi Distr. (Filippov, 2011).

*Othius lapidicola*Märkel & Kiesenwetter, 1848

Tyumen Prov. (Bukhkalo et al., 2011, 2012).

*Othius punctulatus*(Goeze, 1777)

Tyumen Prov. (Bukhkalo et al., 2011, 2012); WS (Schülke, Smetana, 2015).

*Othius subuliformis*Stephens, 1833

Tyumen Prov. (Bukhkalo et al., 2011, 2012); WS (Schülke, Smetana, 2015).

**Tribe** *Xantholinini* Erichson, 1839

**Genus** *Gyrohypnus* Leach, 1819

*Gyrohypnus angustatus*Stephens, 1833

Kemerovo Prov. (Babenko, 1982); Novosibirsk Prov. (Pavlov, 2005); Tomsk Prov. (Babenko, 1982); Tyumen Prov. (Bukhkalo et al., 2011, 2012); WS (Schülke, Smetana, 2015).

*Gyrohypnus atratus*(Heer, 1839)

Kemerovo Prov. (Babenko, 1982); Tomsk Prov. (Babenko, 1982; Babenko, Nuzhnykh, 2014).

*Gyrohypnus fracticornis*O.F. Müller, 1776

Kemerovo Prov. (Babenko, 1982); Novosibirsk Prov. (Pavlov, 2005); Tomsk Prov. (Babenko, 1982); WS (Schülke, Smetana, 2015).

*Gyrohypnus punctulatus*(Paykull, 1789)

Tyumen Prov. (Bukhkalo et al., 2011, 2012); WS (Schülke, Smetana, 2015).

**Genus** *Hypnogyra* Casey, 1906

*Hypnogyra angularis*(Ganglbauer, 1895)

Novosibirsk Prov. (Pavlov, 2005).

**Genus** *Leptacinus* Erichson, 1839

*Leptacinus batychrus*(Gyllenhal, 1827)

Kemerovo Prov. (Babenko, 1982); Novosibirsk Prov. (Pavlov, 2005); Tomsk Prov. (Babenko, 1982; Babenko, Nuzhnykh, 2014); WS (Schülke, Smetana, 2015).

*Leptacinus formicetorum*Märkel, 1841

Novosibirsk Prov. (Pavlov, 2005); WS (Schülke, Smetana, 2015).

*Leptacinus pusillus*(Stephens, 1833)

WS (Schülke, Smetana, 2015).

*Leptacinus sulcifrons*(Stephens, 1833)

Novosibirsk Prov. (Pavlov, 2005); Tyumen Prov. (Bukhkalo et al., 2011, 2012).

**Genus** *Megalinus* Mulsant & Rey, 1877

*Megalinus glabratus*(Gravenhorst, 1802)

Novosibirsk Prov. (Pavlov, 2005).

**Genus** *Nudobius* Thomson, 1860

*Nudobius lentus*(Gravenhorst, 1806)

Kemerovo Prov. (Babenko, 1982); Novosibirsk Prov. (Pavlov, 2005); Tomsk Prov. (Babenko, 1982; Babenko, Nuzhnykh, 2014; Kercheva et al., 2022); WS (Schülke, Smetana, 2015).

**Genus** *Xantholinus* Dejean, 1821

*Xantholinus dvoraki*Coiffait, 1956

WS (Schülke, Smetana, 2015).

*Xantholinus laevigatus*Jacobsen, 1849

Tyumen Prov. (Bukhkalo et al., 2011, 2012); WS (Schülke, Smetana, 2015).

*Xantholinus linearis*(A. G. Olivier, 1795)

Kemerovo Prov. (Babenko, 1982); Novosibirsk Prov. (Pavlov, 2005); Tomsk Prov. (Babenko, 1982); WS (Schülke, Smetana, 2015).

*Xantholinus longiventris*Heer, 1839

Kemerovo Prov. (Babenko, 1982); Tomsk Prov. (Babenko, 1982); WS (Schülke, Smetana, 2015).

*Xantholinus tricolor*(Fabricius, 1787)

Kemerovo Prov. (Babenko, 1982); Novosibirsk Prov. (Pavlov, 2005); Tomsk Prov. (Babenko, 1982; Babenko, Nuzhnykh, 2014); Tyumen Prov. (Bukhkalo et al., 2011, 2012); WS (Schülke, Smetana, 2015).

**References**

Babenko A (1982) Fauna i biotopicheskoe raspredelenie korotkonadkrylye zhukov (Coleoptera, Staphylinidae) v yuzhnoy chasti lesnoy zony zapadnoy Sibiri  Poleznyie i vrednyie nasekomyie Sibiri. Nauka, Novosibirsk, 52-59.

Babenko A, Klass A, Bokova U (2018) Peculiarities of rove beetles species diversity (Coleoptera, Staphylinidae) in agroecosystems of the taiga zone of western Siberia [in Russian, English abstract] [abstract]. TSU, Tomsk 10: 74-76.  <https://elibrary.ru/item.asp?id=35596916>

Babenko A, Nuzhnykh S (2014) Fauna and seasonal dynamics of activity of ground beetles on the berry plantations of Siberian Botanical Garden experimental plot. 2. Fauna and seasonal dynamics of activity of rove beetles (Coleoptera: Staphylinidae) [in Russian, English abstract]. Vestnik Tomskogo gosudarstvennogo universiteta. Biologiya 25(1): 97-110.

Bukhkalo S, Galich D, Sergeeva E, Alemasova N (2011), 110-169 Summary of the beetle fauna of the southern taiga of West Siberia (in the basin of the lower Irtysh river). KMK Scientific Press, Moscow, 267 pp.

Bukhkalo S, Galich D, Sergeeva E, Vazhenina N (2014), 184-185. Summary of the invertebrates fauna of the southern taiga of West Siberia (in the basin of the lower Irtysh river). KMK Scientific Press, Moscow, 189 pp.

Bukhkalo S, Sergeeva E, Semenov V (2012) Fauna zhu-kov-stafilinid (Coleoptera, Staphylinidae) tsentral'noy chasti yuzhnoy taygi Zapadnoy Sibiri [Rove-beetle fauna (Coleoptera, Staphylinidae) of the central part of the southern taiga in West Siberia, Russia]. Evraziatskiy entomol. zhurn.[Euroasian Entomological Journal] 11(4): 343-353.

Filippov I (2011) Annotated list of animals of Khanty-Mansi Distr. [Electronic resource] .107 pp. <https://www.researchgate.net/publication/326367905>

Kercheva A, Babenko A, Krivets S, Krivosheina M, Smirnov N, Tselikh E (2022) Contribution to the Fauna and Bionomics of Entomophagous Insects Feeding on the Small Spruce Bark Beetle *Ips amitinus* (Eichh.) (Coleoptera, Curculionidae: Scolytinae) in West Siberia. Entomological Review 102(4): 432-445. <https://doi.org/10.1134/S0013873822040029>

Krasutsky B (1995) Fungicolous Coleoptera inhabitants of the wood-destroying fungi in the forests of West Siberia [in Russian, English subtitle]. Entomol 74(3): 542-550.

Krasutsky B (1997) Fungicolous Coleoptera inhabiting main wood-destroying fungi in the middle taiga subzone in west Siberia [in Russian]. Entomol 76(4): 770-776.

Krasutsky B (2005), 19-20 Mycetophilic Coleoptera of the Urals and Trans-Urals. Volume 2. System "Mushrooms-insects". Russian Entomological Society, Chelyabinsk, 213 pp.  <https://www.zin.ru/Animalia/coleoptera/rus/krasut05.htm>

Ol’shvang V (1992) Structura I dinamika naseleniya nasekomyh Uzhnogo Yamala.  The science. Ural.department, Ekaterinburg, 103 pp. <https://www.zin.ru/animalia/COLEOPTERA/rus/svpdjv61.htm>

Pavlov E (2002) Contribution to the fauna of rove beetles (Coleoptera, Staphylinidae) of Novosibirsk Oblast [in Russian, English abstract]. Euroasian Entomological Journal 1(1): 67-69.

Pavlov E (2005) The rove-beetles (Coleoptera, Staphylinidae) of the West Siberian forest-steppe zone [in Russian, English abstract]. Euroasian entomological journal 4(3): 223-230.

Pavlov E (2006) Rove beetles (Coleoptera, Staphylinidae) in the northern forest-steppe of the Barabinskaya lowland. Euroasian Entomological Journal 5(3): 206-208.

Ryvkin A (2007) A review of *Lathrobium* species of the *sibiricum* group (Insecta: Coleoptera: Staphylinidae: Paederinae). Bulletin de l’Institut royal des Sciences naturelles de Belgique, Entomologie 77: 179-234.

Ryvkin A (2011) On new and poorly known *Lathrobium* (s.str.) species from Siberia and the Russian Far East (Insecta: Coleoptera: Staphylinidae: Paederinae). Baltic J. Coleopterol. 11(2): 135-170.

Ryvkin A (2012) New species and records of *Stenus* (*Nestus*) of the *canaliculatus* group, with the erection of a new species group (Insecta: Coleoptera: Staphylinidae: Steninae). European Journal of Taxonomy 13: 1-62. <http://dx.doi.org/10.5852/ejt.2012.13>

Ryvkin A (2014) *Stenus (Nestus) pluvius* sp. n., with notes on some related species (Coleoptera: Staphylinidae: Steninae). Acta Biol. Univ. Daugavp 14(2): 187-205.

Schülke M, Smetana A (2015) Staphylinidae, 304–1134 In: Löbl I, Löbl D (Eds) Catalogue of Palaearctic Coleoptera.Volume 2. Hydrophiloidea–Staphylinoidea, Revised and updated edition. Brill, Leiden, Boston, 1702 pp.

Shavrin A (2021) On the Acidota Stephens fauna of Russia (Staphylinidae, Omaliinae, Anthophagini). Journal of Insect Biodiversity 22(1): 1-21. 10.12976/JIB/2021.22.1.1

Shavrin A (2021) On the genus Mannerheimia Mäklin, 1880 (Coleoptera: Staphylinidae: Omaliinae: Anthophagini), with taxonomic and faunistic notes on some species. Zootaxa, 5040(3): 301-333. <https://doi.org/10.11646/zootaxa.5040.3.1>

Smetana A, Shavrin A (2018) Contribution to the knowledge of the genus *Quedius* STEPHENS, 1829 of Siberia and Russian Far East (Coleoptera: Staphylinidae: Staphylinini: Quediina). Linzer biol. Beitr. 50(1): 825-836.

Vazhenina N (2019) Population dynamics for herpetobiont beetles (Coleoptera) in the floodplain of a small tributary in the lower reaches of the Irtysh [in Russian, English abstract]. Amurian Zoological Jornal 11(4): 314-326. <https://cyberleninka.ru/article/n/dinamika-naseleniya-gerpetobiontnyh-zhestkokrylyh-coleoptera-v-poyme-malovodnogo-pritoka-nizhnego-techeniya-reki-irtysh>

Zinchenko V (2003) The coprophilous and nidicolous species structure of Coleoptera from marmots' holes on the territory of Kemerovo Oblast [in Russian, English subtitle]. Euroasian Entomological Journal 2(4): 279-280

Zinovyev E, Akopyan E (2013) Fauna of Coleoptera (Insecta, Coleoptera) of the Berezovskoye reserve (lower Ob). Vestnik Altaiskogo gosudarstvennogo agrarnogo universiteta. Ecology 2(100): 60-66. <https://cyberleninka.ru/article/n/fauna-napochvennyh-zhestkokrylyh-insecta-coleoptera-beryozovskogo-zakaznika-nizhnee-priobie>

Zinovyev E, Borodin A, Trofimova S, Sheinkman V, Rusakov A, Sedov S, Bobkov R, (2016) Late Pleistocene insect complexes (Vakh river, West Siberia, Russia) and their paleoenvironmental charactristics [in russian]. Euroasian Entomological Journal 15(5): 483–498
